# Targeted Infection Control and Tissue Integration via pH-Sensitive Smart Coatings on Implant Surfaces

**DOI:** 10.1021/acsami.5c19344

**Published:** 2025-12-23

**Authors:** Marta Maria Alves Pereira, Rodolfo Debone Piazza, Paula Aboud Barbugli, Oya Tagit, Jeroen J.J.P. van den Beucken, Abhijna Das, Cleyton Alexandre Biffe, Valentim Adelino Ricardo Barão, Stéfany Barbosa Alves da Cruz, Edilson Ervolino, Leonardo Perez Faverani, Vinicius Franzao Ganzaroli, Daniela Leal Zandim-Barcelos, Magda Feres, Belén Retamal-Valdes, Denise Madalena Palomari Spolidorio, Ana Claudia Pavarina, Amanda Paino Santana, Beatriz Severino Verza, Rodrigo Fernando Costa Marques, Rafael Scaf de Molon, Erica Dorigatti de Avila

**Affiliations:** † Department of Dental Materials and Prosthodontics, School of Dentistry, 28108 São Paulo State University (UNESP), Araraquara, São Paulo 14801-903, Brazil; ‡ Postgraduate Program of Dentistry, Federal University of Piauí, Teresina, Piauí 64049-550, Brazil; § Department of Analytical Chemistry, Physical Chemistry and Inorganic, Institute of Chemistry, São Paulo State University (UNESP), Araraquara, São Paulo 14800-060, Brazil; ∥ Institute of Chemistry and Bioanalytics, School of Life Sciences, 537298 University of Applied Sciences and Arts Northwestern Switzerland, Muttenz 4132, Switzerland; ⊥ Dentistry - Regenerative Biomaterials, Radboudumc, Philips Van Leydenlaan 25, Nijmegen 6525 GA, The Netherlands; # Brazilian Nanotechnology National Laboratory (LNNano), 215006 Brazilian Center for Research in Energy and Materials (CNPEM), Campinas, São Paulo 13083-970, Brazil; ∇ Department of Prosthodontics and Periodontology, Universidade Estadual de Campinas (UNICAMP), Faculdade de Odontologia de Piracicaba (FOP), Piracicaba, São Paulo 13414-903, Brazil; ○ Department of Diagnosis and Surgery, School of Dentistry, 207339 São Paulo State University (UNESP), Araçatuba, São Paulo 16015-050, Brazil; ◆ Department of Basic Sciences, School of Dentistry, São Paulo State University (UNESP), Araçatuba, São Paulo 16018-805, Brazil; ¶ Department of Oral Diagnosis, Division of Oral and Maxillofacial Surgery, Piracicaba Dental School, 245124 University of Campinas (UNICAMP), Piracicaba, São Paulo 13414-903, Brazil; △ Department of Diagnostic and Surgery, School of Dentistry, São Paulo State University (UNESP), Araraquara, São Paulo 14801-903, Brazil; ▲ Department of Oral Medicine, Infection, and Immunity, Harvard School of Dental Medicine, Boston, Massachusetts 02115, United States; ▽ Department of Periodontology, RS Master Saude, Aruja, São Paulo 07400445, Brazil; ▼ Department of Physiology and Pathology, School of Dentistry, 153998 São Paulo State University (UNESP), Araraquara, São Paulo 14801-903, Brazil

**Keywords:** drug delivery system, stimuli-responsive biomaterial, antimicrobial coating, smart coating, implant

## Abstract

A stimuli-responsive drug delivery coating is proposed for titanium (Ti) implants to locally treat infectious and peri-implant inflammatory diseases. The system integrates a pH-responsive film based on poly­(methacrylic acid) (PMAA) film over a layer-by-layer (LbL) drug delivery platform containing tetracycline (TC) complexed with anionic beta cyclodextrin (βCD). This smart coating was designed to control drug release, maintain antimicrobial activity against biofilm-forming pathogens, and enhance soft tissue sealing at the implant interface. The coating effectively regulated drug release, exhibited favorable hydrophilicity, and reduced surface roughness compared with untreated Ti surfaces. Cytocompatibility was confirmed in both monolayer cell cultures and collagen matrix environments, with no cytotoxic effects observed up to 6 days. Atomic force microscopy (AFM) revealed enhanced interactions between the PMAA film and cellular components, as evidenced by filopodial projections at the cell margins. The coating’s antibacterial properties were validated using human saliva-derived biofilms, demonstrating broad-spectrum antimicrobial activity against pathogens typically involved in dental implant infections. *In vivo*, a rat subcutaneous tissue model was used to evaluate the immune response. The LbL/TCβCD/PMAA coating significantly reduced inflammation, increased collagen deposition, and elevated CD206 expression, indicating a shift toward an anti-inflammatory and tissue-repair phenotype. This stimuli-responsive coating represents a promising strategy for localized infection control, drug delivery, and soft tissue integration on implant surfaces.

## Graphical abstract

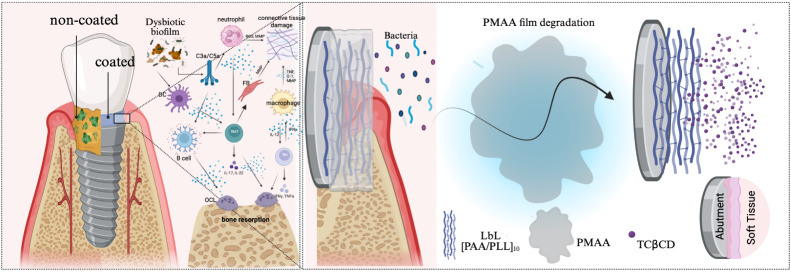

## 1 Introduction

According to the World Health Organization (WHO), severe periodontal diseases are estimated to affect more than 1 billion cases worldwide (https://www.who.int/news-room/fact-sheets/detail/oral-health). Importantly, a patient’s history of periodontitis represents a significant risk factor for peri-implantitis. The use of dental implants is growing fast, especially in high-income countries, which raises concerns about peri-implant diseases, such as peri-implant mucositis and peri-implantitis. Peri-implantitis clearly poses a growing public health problem due to its high prevalence and the dreaded associated consequences that might transcend implant and implant-supported prosthesis loss. As a chronic inflammatory condition, during disease progression the environmental and biological factors in a susceptible host might activate immune and non-immune cells. A greater inflammatory response to peri-implant plaque accumulation could evoke a stronger systemic host response by increased levels of matrix metalloproteinase 8 (MMP-8) and proinflammatory cytokines.^1^

Peri-implantitis is indeed considered the major inflammatory concern in advanced dental care. Although dental implants exhibit high initial success rates for osseointegration (up to 98%)^2^ they yet remain vulnerable to late-stage biological complications.^3,4^ Recent studies indicate a peri-implantitis prevalence ranging from 26.7% to 56% at the patient level after approximately 7.8 years of functional loading.^5^ This means that between a quarter and over half of patients with dental implants may experience peri-implantitis after this time frame. Additionally, disturbing outcomes revealed that costs related to implant complications added up to 557 € for single restorations and 769 € for full arches, pointing out that implant loss was the costliest complication.^6^ The alarming prevalence of peri-implantitis and the high impact on a country’s economy highlight that current clinical procedures aimed at preventing and treating peri-implantitis are insufficient,^7^ and also indicate the need for new strategies to fight the infectious-inflammatory disease.

Managing implant-related infections remains a considerable clinical challenge, requiring a multifaceted approach to control infection, reduce inflammation, and promote tissue regeneration.^8^ Once established, biofilms act as a persistent source of infection and inflammation, leading to peri-implant tissue destruction and implant failure. Although debridement and surface decontamination are integral parts of peri-implantitis management,^9^ their efficacy is limited by the inability to completely remove or inactivate biofilm bacteria due to the microtopography and roughness of implant surfaces.^10^ Therefore, rather than relying solely on surgical decontamination, current research has shifted toward developing bioactive and antimicrobial surface modifications capable of preventing or disrupting biofilm formation at the implant–tissue interface.

In this context, the development of smart coatings that can respond to changes in the peri-implant microenvironment, such as pH fluctuations associated with infection, has emerged as a promising strategy.^11^ Controlled drug release technologies, such as layer-by-layer (LbL) systems,^10,12^ can achieve high local drug concentrations and sustained antimicrobial effects, thereby reducing bacterial recolonization and inflammation. These stimuli-responsive materials can be designed to react to pH, temperature, ionic strength, or specific enzymatic activity, enabling the controlled and localized release of therapeutic agents.^13^

Despite the advantages of LbL, challenges related to the coating development process have slowed progress toward clinical translation.^11^ While some studies have demonstrated the efficacy of LbL-coated implants in preclinical trials,^12^ limitations hindering clinical advancement remain. One significant challenge arises from the inherent differences in properties between the drug and the coating materials, which can lead to a rapid release of a large portion of the drug, particularly within the first 24 h after application,^13^ potentially resulting in suboptimal treatment outcomes. To ensure therapeutic efficacy, it is crucial to achieve a drug concentration capable of sustaining release, ideally for at least 7 days. However, achieving such a sustained release while maintaining a safe concentration presents another challenge, as high drug concentrations can evoke localized toxicity in tissue surrounding the device, compromising both patient safety and treatment effectiveness.^14^

Our preliminary studies have validated a method for drug incorporation within an LbL system on Ti substrates, comprising alternating polyelectrolyte layers of poly­(acrylic acid) (PAA) and poly­(l-lysine) (PLL), and the incorporation of tetracycline (TC) with anionic beta cyclodextrin (βCD).^13,15^ TC was incorporated into the coating for its antimicrobial activity to prevent bacterial colonization on implant surfaces, while βCD serves as a drug carrier, forming inclusion complexes with TC that enhance drug retention and modulate release kinetics.^16^ The succeeded LbL/TCβCD coating was shown to be readily tunable to different physiological scenarios, with a controlled release of the drug up to 30 days.^15^

Building upon these findings, we developed a pH-sensitive poly­(methacrylic acid) (PMAA)-based film as an additional layer to refine and optimize the LbL/TCβCD coating. PMAA was selected due to its synthetic versatility, intrinsic pH responsiveness, and potential to promote soft-tissue compatibility.^16–18^ While PMAA is known to exhibit acidic behavior and a strong negative charge,^19,20^ its modification within our system was carefully engineered to balance charge interactions and enhance epithelial cell adhesion without altering cell morphology. This process required iterative optimization to overcome its inherent acidity while maintaining the functional responsiveness of the coating.

Thus, the PMAA layer was designed to (1) reduce contact toxicity arising from high TC concentrations, (2) sustain and modulate TC release, (3) maintain antimicrobial activity by preventing biofilm recolonization, and (4) enhance soft-tissue sealing around the implant interface, which represents a novel advancement over previously reported TC/βCD systems. From a clinical perspective, this PMAA-modified LbL system was conceived to coat screw-retained abutments, components that can be replaced in established disease cases, representing a clinically translatable, smart antimicrobial coating to manage early peri-implantitis. We hypothesize that the incorporation of a PMAA layer onto TCβCD-modified Ti surfaces will enable controlled, pH-responsive drug release, reducing cytotoxicity while enhancing antimicrobial and soft-tissue integration outcomes.

## 2 Methods and Materials

### 2.1 Materials and Chemical Preparation

Pure titanium (Ti; grade 2, as per the American Society for Testing Materials) machined discs (12 mm in diameter and 1.5 mm thickness) were acquired from Machinefabriek G Janssen B.V. (Valkenswaard, The Netherlands) to simulate commercially available Ti implant abutment surfaces. Polyethylenimine (PEI, m.w. = 25 000 g/mol), poly l-lysine (PLL, m.w. = 15 000–30 000 g/mol), tetracycline hydrochloride (TC, m.w. = 480.90 g/mol), UltraPure^TM^ Tris buffer (≥99.9%), sodium acetate (m.w. = 82.03 g/mol), β-cyclodextrin (βCD), epichlorohydrin (ECH), chloroacetic acid (CAA), allylamine (m.w. = 57.09 g/mol), 1,2-Bis­(dimethylamino)­ethane (TEMED, m.w. = 116.20 g/mol), and methacrylic acid (MAA, m.w. = 86.09 g/mol) were purchased from Sigma-Aldrich (St. Louis, MO, USA). Poly­(acrylic acid) (PAA, m.w. = 60 000 g/mol) and sodium chloride were obtained from Polysciences (Warrington, PA, USA) and Millipore Corporation (Burlington, MA, USA), respectively.

### 2.2 Synthesis of Anionic Beta Cyclodextrin (βCD)

Anionic βCD was synthesized via a one-step condensation reaction using a molar ratio of βCD, epichlorohydrin (EP), and chloroacetic acid (1:10:2).^15^ βCD was dissolved in a 22% NaOH solution and stirred for 24 h, after which CAA was added, and the mixture was heated to 30 °C. EP was quickly added, and the reaction continued for 3.5 h before being stopped with acetone. The acetone was then removed, and the solution was neutralized with hydrochloric acid and dialyzed for 4 days to eliminate unreacted components. The final product was obtained by evaporating the solution, triturating with acetone, and drying under a vacuum.

### 2.3 Synthesis of TC/Anionic βCD

Inclusion complexes of TC with anionic βCD were prepared using a coprecipitation method in a 1:1 molar ratio.^15^ TC was dissolved in water, while anionic βCD was dissolved in heated water at 60 °C. The βCD solution was added dropwise to the TC solution, heated for one h, and stirred overnight at room temperature. The resulting mixture became turbid, indicating the formation of an inclusion complex. The complexes were filtered, washed to remove uncomplexed βCD, and vacuum-dried. The incorporation efficiency was subsequently assessed.^15^

### 2.4 Synthesis of Poly­(methacrylic acid) (PMAA)-Based Film

A PMAA-based polymer was synthesized through the copolymerization of methacrylic acid (MAA) with acrylic acid (AA) and allylamine (Allyl) in a molar ratio of 1:1:2. The resulting copolymer was dialyzed against ultrapure water using a dialysis bag with a cutoff of 3500 Da and was kept overnight under freezing conditions before undergoing lyophilization. A PMAA copolymer solution was prepared at a concentration of 50 mg/mL for further deposition onto the substrate.

### 2.5 Characterization of βCD, TC/βCD, and PMAA

Nuclear magnetic resonance spectroscopy (NMR, Bruker, Bremen, Germany) was used to determine the structure of the PMAA copolymer and to confirm the successful synthesis of anionic βCD. Signals from 1H and 13C experiments were recorded using a spectrometer (Bruker Avance) operated at 600 MHz and 25 °C, with 1 mM samples of βCD, anionic βCD, and PMAA dissolved in deuterated D2O solvent (Figure S1A,B). To confirm TC incorporation into βCD, Fourier transform infrared spectroscopy (FTIR) was performed (Figure S1C). The FTIR measurements were also conducted using a PerkinElmer Dual Frontier spectrometer equipped with an attenuated total reflectance (ATR) accessory featuring a zinc selenide crystal, with a resolution set at 4 cm^–1^ and 128 scans. The reproducibility of the PMAA-based film synthesized in two separate instances was also confirmed (Figure S1D).

### 2.6 TC/Anionic βCD/PMAA-Based Film on LbL-Coated Ti Discs

Prior to LbL coating assembly, Ti discs were cleaned with acetone, followed by Milli-Q water and 2-propanol, and then air-dried for 24 h. To enhance Ti-PEI interaction, the discs were exposed to UV light for 20 min. LbL coatings were then assembled via electrostatic interactions by alternating layers of PAA and PLL, with immersion and washing steps to form a [PAA/PLL]_10_ system (LbL), as previously described.^15^ A 5 mg/mL TCβCD solution was applied to the final layer and statically incubated at 37 °C for 7 days to promote drug diffusion.

PMAA copolymer powder was prepared, and 150 μL of a 50 mg/mL PMAA solution was dropped onto the samples and left to air-dry inside a laminar flow hood for 24 h. After 48 h, the samples were washed with a sodium bicarbonate solution to neutralize acidity, followed by three additional washes with Milli-Q water. Samples were air-dried for another 24 h. The multilayers assembled on Ti discs using PAA and PLL, as established in previous studies, will be referred to as LbL. Experimental controls included Ti, PMAA-based films, and LbL on Ti discs, while experimental samples included LbL/TCβCD, LbL/PMAA, and LbL/TCβCD/PMAA. Group classifications varied based on the method applied.

### 2.7 Coating Characterization and Stability Assessment

Zeta (ζ) potential measurements were conducted to assess the surface charge of the PMAA-based film, aiding in understanding its electrostatic interactions with biological environments. This was performed using an electrokinetic analyzer (SurPASS, Anton Paar GmbH, Austria). Experimental controls included Ti, LbL, and Ti/PMAA, while experimental samples included LbL/PMAA and LbL/TCβCD/PMAA, which were fixed in an adjustable gap cell with a 0.001 mol/L sodium chloride solution (pH = 7.4) as the electrolyte. The experiment was conducted in triplicate.

The wettability of the PMAA-based film was evaluated using contact angle measurements with a sessile drop technique (Dataphysics goniometer, model OCA20), comparing LbL/PMAA on Ti discs against the Ti controls. The integrity of the PMAA-based film was assessed after immersion in phosphate-buffered saline (PBS) and sodium acetate (SA) at different time points (1, 3, 7, and 15 days) at 37 °C. After each time point, samples were air-dried in a laminar flow hood, and the wettability was measured. Experiments were performed in five replicates to ensure statistical robustness.

Surface roughness was quantified using NanoScope analysis software (Bruker, Bremen, Germany) from ten different regions (2 × 2 μm) of LbL/PMAA samples. Atomic force microscopy (AFM) images were obtained using a Catalyst BioScope (Bruker, Bremen, Germany) coupled to a confocal microscope (TCS SP5II, Leica, Mannheim, Germany) in peak-force tapping mode with silicon nitride cantilevers (nominal spring constant: 0.7 N/m). PMAA-based film behavior was assessed after immersion in PBS and SA at various time points (1, 3, 7, and 15 days). Ti discs were used as commercial controls. Experiments were conducted in duplicate, and data were collected from five independent regions of each sample to account for intrasample variability.

The topography of the PMAA-based film was examined by using a Park NX10 AFM (Park Systems Corp., Suwon, Korea) in peak-force tapping mode. Silicon cantilevers with aluminum coatings (spring constant 2.8 N/m) were used to capture images from three regions (20 × 20 μm) per area. Elemental composition after brushing was analyzed by using X-ray photoelectron spectroscopy (XPS) with a K-alpha spectrometer (Thermo Scientific), focusing on elements such as carbon, nitrogen, oxygen, sulfur, and Ti. Additionally, brushing was performed according to ISO/TR 14569-2:2001 for the wear simulation of dental materials. Experiments were conducted in duplicate, and data were collected from three independent regions of each sample to account for intrasample variability.

From a clinical perspective, as the LbL/PMAA system is designed to coat implant abutment surfaces, its mechanical stability was evaluated through simulated toothbrushing. Samples were brushed with nylon toothbrushes (Tek, Johnson & Johnson, Brazil) using a brushing machine (Elquio Maq Escovacao, São Carlos, Brazil) applying 200 g vertical force at 60 cycles/min.^12^ Each sample group underwent 240 cycles, simulating 1 day of denture hygiene. After brushing, the samples were washed and air-dried, and roughness measurements were taken using the Park NX10 AFM system to assess the impact on the PMAA-based film. XPS was employed to measure the remaining elemental composition after the brushing.

In order to complement our previous analyses and gain a more reliable understanding about the film structure, mass, and thickness, the PMAA-based film was subject to ellipsometry analyses. However, to avoid the effects of irregular surface topography of Ti discs, we assembled a PMAA-based film on silicon substrate samples. The experimental setup was designed by integrating a commercial variable-angle spectroscopic ellipsometer (J.A. Woollam Company) at a variable angle of incidence from 50° to 70°, over the full spectral range from 250 to 1500 nm. The dip-coating method was employed to prepare the samples for ellipsometry thickness measurements.

### 2.8 Standard Solution and System Suitability for Drug Release

To evaluate the release control of TC from PMAA-based films, LbL/TCβCD coatings were compared with LbL/TCβCD/PMAA coatings on Ti discs. Three samples of each type were immersed in 2 mL of PBS (pH = 7.4) and SA buffer (pH = 4.5) at 37 °C. TC release was monitored over 1, 3, 7, and 15 days, with 1 mL of supernatant collected at each time point for analysis, followed by the addition of fresh buffer.

The detection limit for TC was established using standard solutions in PBS and SA buffers, ranging from 0.5 to 250 μg/mL. Released TC/anionic βCD in the supernatants was analyzed via high-performance liquid chromatography (HPLC; Hitachi, Mannheim, Germany), equipped with a pump, UV detector, autosampler, and a LiChrospher RP-18 column, operated at a flow rate of 0.8 mL/min with a 30 μL injection volume.

### 2.9 *In Vitro* Cytotoxicity Tests

#### 2.9.1 Cell Culture and Growth Conditions

HaCaT immortalized human keratinocytes and human gingival fibroblasts (HGF-0089, Rio de Janeiro Cell Bank Code 0089) were cultured in Dulbecco’s Modified Eagle’s Medium High Glucose (DMEM, Sigma Chemical Co., St. Louis, MO, USA), supplemented with 10% fetal bovine serum (FBS, Gibco, Grand Island, NY, USA), 1% penicillin/streptomycin (Sigma-Aldrich, St. Louis, MO, USA), and 2 mM l-glutamine (Gibco, Grand Island, NY, USA), in a humidified atmosphere with 5% CO_2_ at 37 °C. Upon reaching 90% confluence, cells between passages 3 and 8 were washed with PBS, recovered with trypsin solution (Sigma-Aldrich, St. Louis, MO), and resuspended in fresh medium.

#### 2.9.2 HaCaT and HGF Cell Viability

The cytocompatibility of the PMAA-based film was assessed by evaluating the viability, spreading, and metabolic activities of HaCaT and HGF cells. Cells were seeded directly onto LbL/TCβCD/PMAA and LbL/PMAA-coated Ti discs at 2 × 10^5^ cells per well for HaCaT and 1 × 10^5^ cells per well for HGF. After 24 h, samples were stained with Hoechst 33342/propidium iodide (Invitrogen, CA, USA) to assess cell viability using the LIVE/DEAD staining kit (Invitrogen, Carlsbad, CA), following the manufacturer’s instructions. The samples were visualized using a PASCAL LSM 800 Confocal Laser Scanning Microscope (Carl Zeiss, Jena, Germany), and images were acquired with 10× and 40× dry (Plan Neo-Fluar NA 0.3 air) objective lenses. Excitation wavelengths were 405–470 nm and 561–620 nm, respectively. All the cell population was stained in blue, and dead cells were stained in red, and they also performed brightfield acquisition, respectively. Positive controls (C^+^) involved HaCaT cells seeded on polystyrene well plates, while cells exposed to 9% Triton for 5 min served as death controls (C^–^). The experiment was performed in duplicate.

#### 2.9.3 HaCaT Morphology

Changes in the HaCaT cell morphology were examined using scanning electron microscopy (SEM). After 24 h of seeding, samples were fixed with 2.5% glutaraldehyde for 1 h at ∼25 °C, washed with PBS, and dehydrated using graded ethanol solutions (70%, 90%, and absolute ethanol). The samples were left in a vacuum desiccator for 48 h and analyzed using a JSM-6610LV SEM (JEOL, 10 kV, WD 8 mm, and SS30). Negative and positive controls were included.

#### 2.9.4 DNA Integrity

We evaluated whether the PMAA-based film affected DNA integrity or induced degradation. HaCaT cells (2 × 10^5^ cells/well) were cultured on PMAA-based film-coated Ti discs in a 24-well plate under 5% CO_2_ at 37 °C for 24 h. Cells were harvested by gently scraping with a plastic pipet tip into 150 μL of PBS, transferred to 1.5 mL Eppendorf tubes, and subjected to DNA isolation using a MasterPure DNA Purification Kit (EPICENTER). DNA integrity was confirmed via electrophoresis on a 1.0% agarose gel at 60 V for 1 h. DNA bands were visualized and recorded under UV light using a Gel Documentation System (Bio-Rad Laboratories). The experiment was performed in duplicate with two repetitions.

#### 2.9.5 HaCaT Cell Shape Detection by AFM

To further investigate the effect of PMAA-based films on the live cell shape, AFM was used. HaCaT cells were seeded at 1 × 10^5^ cells/well on LbL/PMAA-coated Ti discs and incubated for 24 h under 5% CO_2_ at 37 °C. Cell topography was imaged using QI mode AFM (JPK NanoWizard 4, Bruker Nano GmbH, Germany) with a PFQNM-LC-CAL probe (Bruker Nano GmbH). Cells cultured on Ti discs and Petri dishes served as commercial controls.

#### 2.9.6 HGF Cell Proliferation

HGF cell proliferation on LbL/PMAA and LbL/TCβCD/PMAA-coated Ti discs was quantified using 10% alamarBlue (Invitrogen, CA, USA). Cells were incubated in a humidified atmosphere with 5% CO_2_ at 37 °C for 24 h. Ti discs were used as commercial controls. Cell proliferation was measured using a spectrophotometer (Synergy HTX Multi-Mode Microplate Reader, BioTek, Baden-Württemberg, Germany) at wavelengths of 570 and 600 nm. The experiment was performed in triplicate with three repetitions.

#### 2.9.7 3-D Collagen Cell Morphology, Viability, and Cytokine Secretion

HGF cells were cultured on LbL/PMAA and LbL/TCβCD/PMAA-coated samples placed in a 12-well plate. A collagen solution was prepared using DMEM, FBS, and rat tail type-I collagen (3 mg/mL), and 1 mL of the solution was added to each sample for 24 h at 37 °C in 5% CO_2_. Collagen gel contraction was assessed at 1, 3, and 6 days using a universal caliper. Cell viability was measured using the MTT reduction assay after 6 days of exposure. The absorbance of formazan dye was measured at 562 nm using a spectrophotometer (EZ-Read-400E, Imp Exp LTDA, SP, Brazil). Cytokine quantification was performed using supernatants collected on days 1, 3, and 6, frozen at −20 °C, and analyzed using a BD Cytometric Bead Array Human Inflammatory Cytokines Kit. For cytokine quantification, supernatants were collected on days 1, 3, and 6, frozen at −20 °C, and analyzed using the BD Cytometric Bead Array Human Inflammatory Cytokines Kit to measure IL-1β, IL-6, IL-8, IL-10, IL-12, and TNF-α. After thawing, standards were diluted, and capture beads were added to the samples for incubation. Samples were analyzed using a BD FACSAria Fusion Flow Cytometer (BD Biosciences, San Jose, CA, USA), and data were processed with FCAP Array software v3 (BD Biosciences, San Jose, CA, USA).

### 2.10 Tests for Antibacterial Activity

#### 2.10.1 Human Saliva Culture and Growth Conditions

Saliva was collected from five subjects with peri-implantitis after obtaining ethical approval (Ethics Committee on Human Research of the School of Dentistry at Araraquara—UNESP, protocol number CAAE: 53045321.2.0000.5416). Inclusion criteria included participants aged 30–50 with at least three implants showing ≥3 mm of bone loss on radiographs, along with bleeding on probing and suppuration.^12^ Exclusion criteria ruled out systemic diseases and recent medication use. Patients were instructed not to brush their teeth or eat for 1 h before collection. A total of 10 mL of non-stimulated whole saliva was collected and stored at −20 °C in aliquots for microbial analysis. The pooled saliva was centrifuged to remove debris, and the supernatant was inoculated into a modified rich medium (SHI-FSMS) to support diverse oral taxa,^12^ then incubated under anaerobic conditions. For antimicrobial testing, LbL/TCβCD/PMAA-coated Ti discs were used as experimental samples, while uncoated Ti discs served as the controls. To assess early biofilm formation, an overnight-cultured microbial community was adjusted to an optical density of 0.3 at 600 nm and inoculated onto both the experimental and control discs in sterile 24-well plates. After incubation, the discs were washed with sterile phosphate-buffered saline (PBS) before further analysis.

#### 2.10.2 Bacterial Viability and Morphology Assessment

Antibacterial activity of the LbL/TCβCD/PMAA coating on Ti discs was assessed via a standard colony-forming unit (CFU) analysis. Following PBS washing, the discs were incubated in fresh SHI medium, and early biofilms were harvested for stepwise dilution to quantify viable bacteria. After 3 days of incubation, colony counts on agar plates were compared between the experimental and control Ti discs. To observe the effects of the PMAA-based film on initial biofilm formation, SEM (JEOL JSM-6610LV) was performed after 24 h of incubation. The discs were rinsed with PBS to remove unattached bacteria, fixed with 2.5% glutaraldehyde, and dehydrated using a graded ethanol series. The samples were then vacuum-dried and gold-coated for SEM imaging. Images were taken at ×1k and ×5k magnifications from three different areas of each disc. The experiment was conducted in duplicate to ensure the reliability.

### 2.11 Animal Experiments

Animal experiments were conducted to assess the biocompatibility and safety of the PMAA-based film for use in the LbL coating system. The animal protocol was approved by the Ethical Committee in the Use of Animals from the São Paulo State University, School of Dentistry, Araçatuba, SP, Brazil (#22/2024). Male adult Wistar rats (*Rattus norvegicus*) (500–550 g) were housed in plastic cages within a temperature-controlled environment (21 ± 1 °C), maintained at 65–75% humidity, and subjected to 12-h dark/light cycles. They had *ad libitum* access to food (a normal chow diet) and water. Two experimental groups were used: LbL/PMAA and LbL/TCβCD/PMAA systems, with 7 animals per group. A group composed of implantation with pure Ti discs was also used and served as a control group. Sample size was determined using G Power, targeting an 80% study power and a 5% significance level. The animal experimentation followed all the ARRIVE guidelines^21^ to conduct a study in animals.

#### 2.11.1 Implantation Surgery

The rats were anesthetized with a combination of xylazine (10 mg/kg; Fort Dodge Sade Animal Ltd., Campinas, SP, Brazil) and ketamine (80 mg/kg; Fort Dodge Sade Animal Ltd., Campinas, SP, Brazil). Three 1 cm incisions were made on the rats’ backs, and each animal received three samples (Ti, LbL/PMAA, and LbL/TCβCD/PMAA), randomly distributed. The samples were stabilized in the subcutaneous tissue with sutures (silk 4.0, Ethicon, Johnson Prod., São José dos Campos, Brazil).

At 2 and 7 days postsurgery, the animals were euthanized via anesthetic overdose. Samples containing the discs and surrounding soft tissue were retrieved using punch biopsies and fixed with 4% formaldehyde (Synth, Labsynth Produtos Para Laboratórios Ltda., SP, Brazil) for 48 h. The tissue in contact with the Ti discs was carefully dissected and washed with running water. The samples were then dehydrated with ethanol (Synth), cleared with xylene (Synth), embedded in paraffin (Synth), and sectioned (5 μm thickness) using a Leica RM225 microtome.

#### 2.11.2 Histopathological and Histochemical Analysis of Samples

For histopathological analysis, three equidistant sections of the connective tissue in contact with the central portion of the Ti disc were stained with hematoxylin and eosin (H&E). A blinded and calibrated histologist performed all histopathological analysis using an optical microscope (AxioLab Carl Zeiss, Göttingen, Germany). The following parameters were evaluated: nature and intensity of the local inflammatory response; extent of the inflammatory process; pattern of cellularity and structuring of the connective tissue; and state of the local vasculature.

For the histochemical analysis of the quantity and level of maturation of collagen fibers, three equidistant sections of the previously mentioned region were treated with Picrosirius red. Quantitative and qualitative histochemical analysis of collagen fibers was performed by a certified blinded histologist using polarized light microscopy (DM4000 B, Leica Microsystems, Wetzlar, Germany) in connective tissue in contact with the central part of the Ti disc. In each of the histological sections, a 340 μm x 240 μm image was obtained. In this area, the percentage occupied by the different colors of collagen fibers was measured by using the program QWin (Leica QWin V3, Leica Microsystems). The variation from green to yellow was considered as thin, immature collagen fibers, and the variation from orange to red was considered as thick, mature collagen fibers.

#### 2.11.3 Immunohistochemistry

The immunohistochemical processing followed the protocol described previously by Souza et al.^22^ Briefly, antigen retrieval was performed by immersing the histological sections in citrate buffer within a pressurized chamber (Decloaking Chamber, Biocare Medical, CA, USA). To block endogenous peroxidase and nonspecific sites, 3% hydrogen peroxide (Sigma-Aldrich) for 1 h and 1.5% bovine serum albumin (Sigma-Aldrich) for 12 h were used, respectively. The histological slices were divided into three batches, and each one was incubated for 24 h with one of the following primary antibodies: rabbit CD45 antibody (20103-1-AP, 1:200; Proteintech, Rosemont, IL, USA); rabbit NOS2 antibody (sc649, 1:100; Santa Cruz Biotechnology, Dallas, TX, USA); and mouse CD206 antibody (sc58986, 1:100; Santa Cruz Biotechnology, Dallas, TX, USA). For signal amplification, biotinylated horse antimouse/rabbit IgG antibody (1:100; Vector Laboratories, Newark, CA, USA) was used for 2 h, and streptavidin–HRP (1:100; Vector Laboratories) was used for 2 h. The presence of the HRP enzyme was detected using 3,3′-diaminobenzidine tetrahydrochloride (DAB) (ImmPACT DAB Substrate kit, peroxidase, Vector Laboratories) for 1 min. The histological sections were counterstained with Harris hematoxylin (Sigma-Aldrich). For the negative controls, only the primary antibodies were omitted.

Immunohistochemical analysis was performed by a blinded, certified histologist using an optical microscope (AxioLab Carl Zeiss). For each target, one histological section was selected per animal, and a 340 μm × 240 μm image was obtained in the connective tissue in contact with the central part of the Ti disc. In this area, the number of immunolabeling cells (CD45-positive, NOS2-positive, and CD206-positive) was quantified. The results were expressed by considering the number of immunolabeling cells per field.

### 2.12 Statistical Data Analysis

Data analysis was performed based on data distribution quality. Normality was tested using the D’Agostino & Pearson test. Statistical comparisons were performed by one-way analysis of variance (ANOVA), and Post Hoc tests were employed according to each purpose: Tukey’s to make all of the pairwise comparisons between groups; Sidak’s as a pairwise multiple comparison test; or Dunnett’s to compare a set of experimental groups against a single control mean. GraphPad Prism version 10.1.0 (GraphPad Software, CA, USA) was employed for all statistical analysis. Data are presented as mean ± standard deviation (SD), and statistical significance was set at *p* < 0.05.

## 3 Results and Discussion

The LbL assembly of polyelectrolytes on solid substrates can be applied to create functional thin films on implant abutment surfaces, incorporating drugs under physiological conditions.^12,15^ This method preserves the inherent properties of the drug while enhancing its stability for delivery.^12,16^ The ability to precisely control the thickness of the LbL coating, combined with the polyelectrolytes’ responsiveness to external stimuli, such as temperature, enzymes, light, pressure, and pH, positions the LbL system as a functional platform for a determined period.^15^ In the context of peri-implantitis, this multifunctional and degradable approach may offer an effective strategy for creating antimicrobial surfaces capable of releasing drugs at controlled concentrations over an optimal duration, addressing the disease.^13,15^ Our preliminary data^13,15^ demonstrated the successful incorporation of TC into an LbL system assembled using alternating polyelectrolyte layers of PAA and PLL, along with the complexation of TC with anionic βCD. The resulting LbL/TCβCD coating on Ti was shown to be highly tunable to different physiological conditions, with a controlled drug release lasting up to 30 days.^13,15^ Given the importance of soft tissue integration on abutment surfaces and the need to protect TC within the multilayers, this study focused on developing an acidic, pH-sensitive PMAA-based film to cover the LbL/TCβCD coating.

### 3.1 Physicochemical Properties and Stability Assessment of LbL/TCβCD/PMAA

The surface properties of biomaterials, such as roughness, wettability, and surface charge, play a crucial role in determining cell behavior, including morphology and functional responses. Among these properties, surface charge is particularly significant, as it affects the adsorption of proteins, which is essential for cell adhesion.^23,24^ Different cell types require specific material properties, and surface charge can influence the quantity, type, and conformation of these adsorbed proteins.^23^ Positively charged surfaces, for instance, can enhance cell proliferation and spreading by activating immune signaling pathways and regenerative processes during the early stages of cellular responses.^25^ Here, we developed an LbL system on Ti discs using carboxylic acid-terminated poly­(acrylic acid) (PAA) and amine poly-l-lysine (PLL). This combination allows for the iterative assembly of polyelectrolyte layers through alternating immersion of Ti discs in solutions of polycations and polyanions with rinsing steps in between to produce a stable multilayer system.

The surface characterization of the LbL/TCβCD/PMAA system provided insights into the electrostatic interactions governing multilayer formation. As the layers are sequentially assembled, the electrostatic balance is maintained within the structure; however, on the outer surface, charge compensation can be incomplete, generating a net surface charge that modifies the surface zeta potential. In general, surface charges arise from dissociation, adsorption, or protonation of functional groups and can be quantitatively assessed through ζ potential measurements.^25–27^

Zeta potential is the measure of the electrical potential at the shearing plane of a charged surface immersed in a liquid, representing the strength of the electrostatic repulsion or attraction capacity of this surface.^26^ In the context of our study, zeta potential measurements allow us to characterize each stage of the titanium-based implant surface modification, describing the presence of the polyelectrolyte, drug, and PMAA-based film layers. The observed increase in ζ potential compared to Ti controls indicates the presence of uncompensated surface charges, mainly attributed to the positively charged tetracycline cations within the multilayer. This electrostatic imbalance is expected as multilayers form since charge compensation within the film can differ from the outermost layer, leading to a net surface charge.

Our experimental results demonstrated that the ζ potential of LbL films in an aqueous environment was significantly higher than that of Ti controls (Figure 1A), likely due to the presence of positively charged tetracycline (TC) cations within the outermost layers. This electrostatic feature is crucial, as it modulates the adsorption of ionic species, protein–surface interactions, and early biological responses, which are all relevant for implant surface bioactivity and cell adhesion.

**Figure 1:**
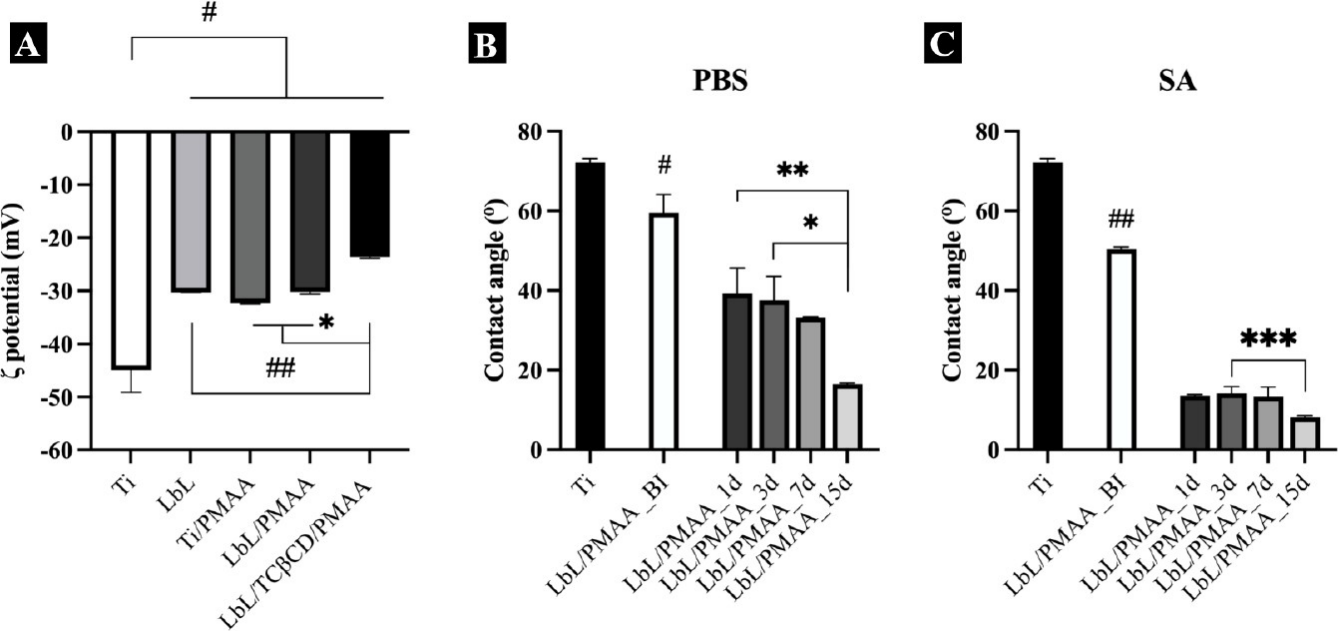
ζ-Potential values of Ti, LbL, PMAA, LbL/PMAA, and LbL/TCβCD/PMAA-coated Ti surfaces before and after immersion in neutral and acidic solutions for 1, 3, 7, and 15 days. The results show a progressive increase in surface charge, with a reduction in negative potential observed in the presence of LbL coatings, particularly after TCβCD incorporation. (A) Statistical comparisons of ζ-potential values were performed by one-way analysis of variance (ANOVA) followed by Tukey’s post hoc test for multiple comparisons (GraphPad Prism v10.10c, GraphPad Software, CA, USA). Data are presented as mean ± standard deviation (SD); statistically significant differences are indicated as #, ##, *p < 0.05. (B,C) The effect of pH on the stability of LbL/PMAA-based films was evaluated by changes in hydrophilicity after immersion in (B) PBS and (C) SA for 1, 3, 7, and 15 days. Contact angle values at each time point were compared with baseline (preimmersion) means using one-way ANOVA with Tukey’s post hoc test. Data are expressed as mean ± SD; statistically significant differences are indicated as #, ##, *, **, ***p < 0.05.

Additionally, understanding the interaction between the PMAA-based film and water is crucial for assessing cell behavior. Clinically, hydrophilic surfaces interact closely with biological fluids, allowing for adequate protein adsorption and subsequent interactions with cell receptors. In contrast, hydrophobic surfaces can trap air bubbles, which may interfere with protein adsorption and cell receptor adhesion or activation. We measured the contact angle of PMAA-coated Ti discs in air and evaluated changes in wettability before (LbL/PMAA_BI) and after immersion in neutral and acidic solutions at different time points: 1, 3, 7, and 15 days. Our findings showed that the PMAA-based film significantly enhanced the hydrophilicity of the Ti surface, which is advantageous for biomedical applications. Notably, after 1 day of immersion in both pH conditions, we observed a marked increase in surface wettability. Although initial changes over the first week were less pronounced, there was a gradual increase in hydrophilicity over time. These alterations in contact angle values confirm the PMAA-based film’s responsiveness to aqueous environments (Figure 1B,C).

The physical properties of implant biomaterial topography, covering porosity and roughness, can influence bacterial adhesion and biofilm formation.^27^ Our results showed that the LbL/PMAA coatings, with or without TCβCD, significantly increased surface roughness compared to (machined) Ti alone. However, these values remain below those required for commercial applications (Figure 2A–D). To assess the stability of the PMAA film, we evaluated the topography and roughness of the PMAA-based coating after immersion in buffers at different pH levels. Samples were incubated for 1, 3, 5, 10, and 15 days, then air-dried and imaged with AFM. The surface roughness was calculated in terms of Ra, and 2 × 2 μm scan size images confirmed the observed film morphology at each time point. The PMAA-based film exhibited a conformational modification that was more pronounced under acidic conditions, with distinct differences in film morphology observed across varying pH levels (Figure 2B,D). For samples immersed in PBS, PMAA-based films maintained their morphology over the entire incubation period (Figure 2A,C). The presence of TCβCD appeared to reduce roughness differences under neutral conditions regardless of the time point.

**Figure 2:**
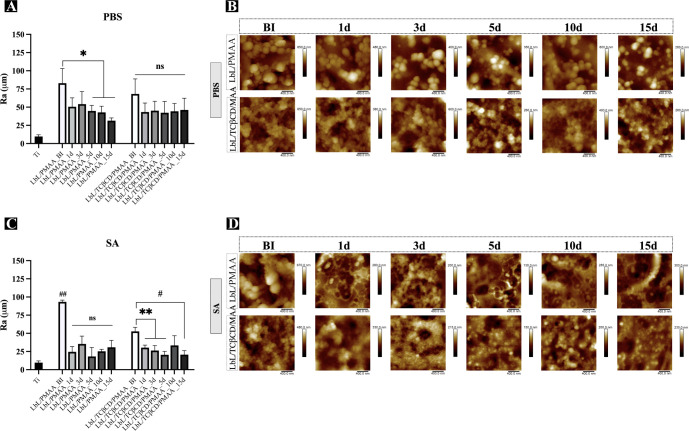
Effect of pH on the stability of LbL/PMAA-based films, evaluated through changes in surface roughness after immersion in (A) PBS and (B) SA for 1, 3, 5, 10, and 15 days. Contact angle values at each time point were compared with baseline (preimmersion) values for each pH condition. Statistical analyses were performed using one-way analysis of variance (ANOVA) followed by Tukey’s multiple comparison post hoc test (GraphPad Prism v10.10c; GraphPad Software, CA, USA). Data are presented as mean ± standard deviation (SD), and statistically significant differences are indicated as *, **, #*p* < 0.05. (C,D) Representative morphological changes of LbL/PMAA and LbL/TCβCD/PMAA coatings under neutral (C) and acidic (D) conditions after 1, 3, 5, 10, and 15 days of incubation.

These findings are consistent with previous research, which has shown that surface modifications can affect both mechanical properties and biological interactions, including antimicrobial activity.^28,29^ High surface roughness can facilitate bacterial accumulation, either through direct chemical interaction or mechanical retention due to surface irregularities, making microbial removal more difficult.^28,29^ It has been reported that a roughness value below 200 nm does not further affect microorganism adherence.^30,31^ Beyond surface effects on osseointegration of dental implants,^32,33^ surface topography has been linked to T-cell hyperplasia due to bacterial colonization on rough breast implants.^34,35^

Moreover, the behavior of the PMAA film under varying pH conditions suggests its potential application in dynamic biological environments where pH fluctuations are common, further supporting the utility of LbL systems in biomedical applications. Indeed, the film presents its pH-sensitive characteristics due to its composition based on poly­(methacrylic acid) (PMAA), a carboxylic acid polymer that exhibits responsive behavior to pH changes.^17^ In environments with a higher pH (typically above 5.5), the carboxylic acid groups deprotonate.^17^ This process introduces negative charges along the polymer backbone. The resulting electrostatic repulsion between the similarly charged polymer chains forces the network to swell, significantly expanding the mesh size and allowing for greater diffusion of molecules.^17^ Conversely, in acidic environments (pH below 5.5), the carboxyl groups become protonated.^17^ This neutralizes the negative charges, dramatically reducing the electrostatic repulsion. Consequently, the PMAA film collapses, the polymer chains adopt a contracted configuration, and the mesh size shrinks, effectively controlling the diffusion and release of the encapsulated molecules. This characteristic reversible transition—collapse in acidic environments and expansion at higher pH levels—is critical. It enables the film to act as a controlled drug delivery system that precisely responds to changes in environmental pH, making PMAA-based films highly suitable for biomedical applications.^36,37^

pH-sensitive film thickness is a critical determinant in the controlled processing and quality assessment for drug delivery. A range of 50–100 nm is often desirable because this thickness provides a balance between surface coverage and responsiveness to stimuli, such as pH or temperature.^38,39^ Here, the PMAA-based film yielded an average thickness of 68.7 ± 3.5 nm, which falls within this typical range for thin polymeric films used in biomedical and coating applications.

The LbL system as an antimicrobial coating represents a viable strategy for treating peri-implantitis through controlled drug delivery.^10^ The system’s responsiveness means that the polyelectrolytes exhibited a conformational modification in response to external stimuli, releasing the incorporated drug in unstable environments.^10^ While our focus has been on the chemical impact of the system, it is also crucial to understand how mechanical actions may affect the degradation process. In this sense, the wear resistance of the PMAA-based film was evaluated through mechanical brushing tests, where we compared images and roughness values of the PMAA-based film before and after brushing (Figure 3 and Table 1, respectively). The results clearly indicate that brushing caused significant detachment of the coating, demonstrating that routine mechanical actions, such as brushing, can accelerate film degradation. This finding suggests that although the PMAA-based film provides controlled drug release and biocompatibility, its mechanical stability under dynamic oral conditions remains a challenge.

**Table 1:** Surface Roughness (Sq) Values of PMAA-Based Films before and after Mechanical Brushing Demonstrate the Effect of Mechanical Stress on Film Stability

RMS roughness (sq):	Ti	LbL	PMAA	LbL/PMAA	LbL/TCβCD/PMAA
**Before toothbrushing**	116.9	156.9	100.2	141	166.9
	148.2	179.2	95.93	172.3	122.5
	135.3	161.5	90.74	153	89.68
**Mean**	133.5	165.9	95.6	155.4	126.4
**StD**	15.7	11.8	4.7	15.8	38.8
**After toothbrushing**	154	118.9	121.6	151.9	235.1
	117	170.6	117.9	150.2	211.9
	132.2	183	132.2	181.1	158.5
**Mean**	134.4	157.5	123.9	161.1	201.8
**StD**	18.6	34.0	7.4	17.4	39.3

**Figure 3:**
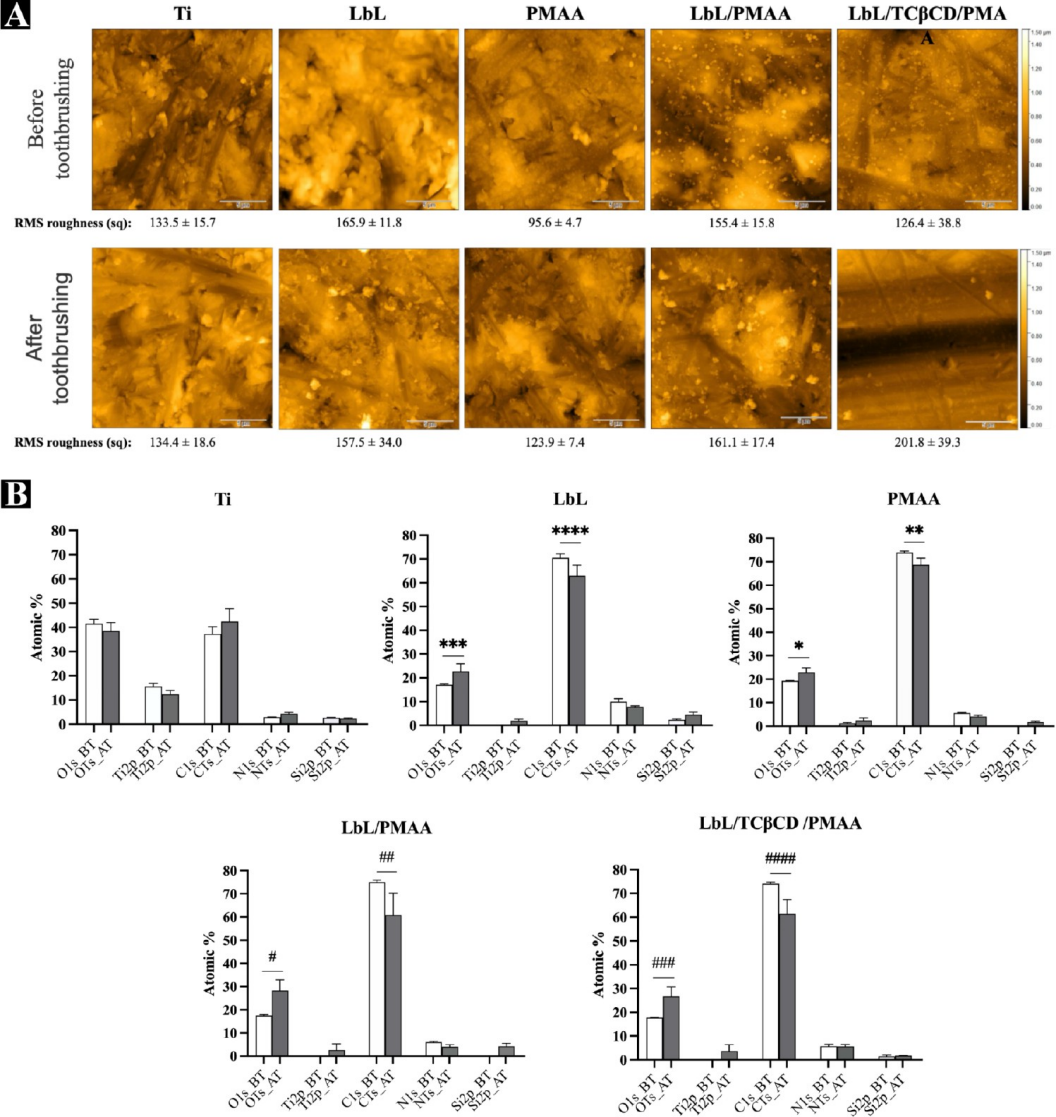
Evaluation of the effect of mechanical brushing on the stability of PMAA-based films and the contribution of each system component to degradation resistance. Morphological (A) and chemical composition (B) analyses were performed before (BT) and after toothbrushing (TB). Statistical analyses were conducted using one-way analysis of variance (ANOVA) followed by Sidak’s multiple comparisons post hoc test in GraphPad Prism version 10.10c (GraphPad Software, CA, USA). Data are presented as mean ± standard deviation (SD). Statistically significant differences are indicated as *, **, ***, ****, #, ##, ###, #### (*p* < 0.05).

In fact, to improve the mechanical stability of the LbL/TCβCD/PMAA coating, a mild cross-linking approach was implemented during synthesis. Mechanical brushing tests (Figure 3) showed partial detachment under simulated oral conditions, indicating a moderate film resilience. Initially, the use of water as a solvent led to rapid degradation; therefore, 1% glutaraldehyde was introduced to promote limited cross-linking between the amine groups of poly­(l-lysine), resulting in enhanced cohesion without compromising cytocompatibility. Importantly, the system was not designed to be completely inert but intentionally pH-responsive and biodegradable, allowing gradual conformational changes and controlled drug release over time. This responsiveness is an inherent feature of LbL assemblies, which rely on electrostatic interactions, relatively weak and reversible by nature.^10^ Such behavior is desirable, since the coating is being developed for clinical situations where peri-implant disease is already established, and therefore a controlled release of the incorporated drug is required, acting to treat the disease as a direct approach.^40^

Thus, while we made efforts to slightly improve the coating’s stability through optimized synthesis conditions, we maintained its stimuli-responsive and biodegradable character, which is essential for its intended antimicrobial and therapeutic function.

A previous study has underscored the significant role of mechanical forces on the stability of surface coatings, revealing that regular oral hygiene practices can impact the durability of antimicrobial surfaces.^27^ Our findings reinforce the need for further investigation into the design of LbL coatings that can withstand mechanical abrasion while preserving their drug delivery capabilities. Optimizing these coatings to endure the mechanical forces common in the oral cavity is essential for enhancing their clinical effectiveness. Understanding the interplay between mechanical forces and the chemical responsiveness of LbL systems is crucial for developing robust coatings. These coatings must not only deliver sustained antimicrobial effects but also endure the rigorous conditions of daily oral care, ensuring long-term efficacy and safety in preventing peri-implantitis and other related conditions.

### 3.2 PMAA-Based Film Controls TCβCD Release from LbL System

Consistent with our previous findings,^15^ our results demonstrate a burst release of TCβCD from the LbL system within the first 3 days of incubation, with a more robust release observed in acidic conditions, simulating an inflammatory environment. Notably, a sustained release of TCβCD was evident over the initial 7 days (Figure 5A). The presence of the PMAA-based film on the LbL/TCβCD system significantly controlled drug release, acting as a barrier layer.

The burst release phenomenon is marked by the swift desorption of drug molecules from the surface of the polymer film during the initial release experiment. It is important to note that during the manufacturing process, some of the drug may become trapped or weakly adsorbed to the surface of the polymer matrix. This fraction is promptly released upon contact with the biological fluid. Upon exposure to an aqueous environment, these superficially bound TCβCD molecules are quickly released, providing an immediate high local concentration of the antimicrobial agent. This initial release is followed by a more sustained release phase, governed by diffusion through the PMAA matrix and, in part, by the pH-responsive swelling behavior of the polymer. The pH sensitivity of PMAA ensures that in acidic microenvironments, where biofilms often create local pH drops, the polymer collapses and slows further release, whereas at neutral pH, the polymer expands, allowing controlled diffusion of the drug over time. This combination of burst and sustained release maximizes the early antimicrobial efficacy while maintaining prolonged protection against microbial recolonization. The burst release of TCβCD occurs mainly due to the hydrophilic nature of tetracycline, which causes a portion of the drug to be released rapidly during the first 24 h. In our previous study, we observed an initial release of approximately 400 μg/mL, a concentration that was not cytotoxic according to extract tests.^15^ This initial burst happens because the hydrophilic tetracycline molecules located near the surface of the system tend to diffuse quickly into the surrounding medium.^13^ However, the incorporation of an ionic βCD helped to reduce the extent of this burst. The βCD forms inclusion complexes with tetracycline, improving drug retention within the system and allowing a more sustained release profile over time.^15^ Confocal microscopy confirmed that part of the drug remains entrapped inside the βCD structure, which explains the slower release after the first 24 h and the maintenance of effective, nontoxic concentrations for several days.^15^

In acidic conditions, a slightly higher concentration of TCβCD was detected over time in samples coated with the PMAA-based film (Figure 4A). Conversely, under neutral pH conditions, which simulate a healthy environment, the TCβCD release from the PMAA-coated samples remained relatively steady and low between days 1 and 15 (Figure 4A). The ability of the PMAA-based film to retain the drug within the LbL system raises important questions about the persistence of the antibacterial activity beneath the polymeric film. These observations indicate that the PMAA-based film not only acts as a barrier but also plays a critical role in retaining the release kinetics of TCβCD. Previous research supports that polymeric coatings can significantly influence drug release profiles, particularly in response to environmental stimuli.^33,41,42^ The enhanced release in acidic conditions suggests that inflammation can be effectively targeted with controlled drug delivery systems, potentially improving therapeutic outcomes for peri-implantitis treatment.^15^

**Figure 4:**
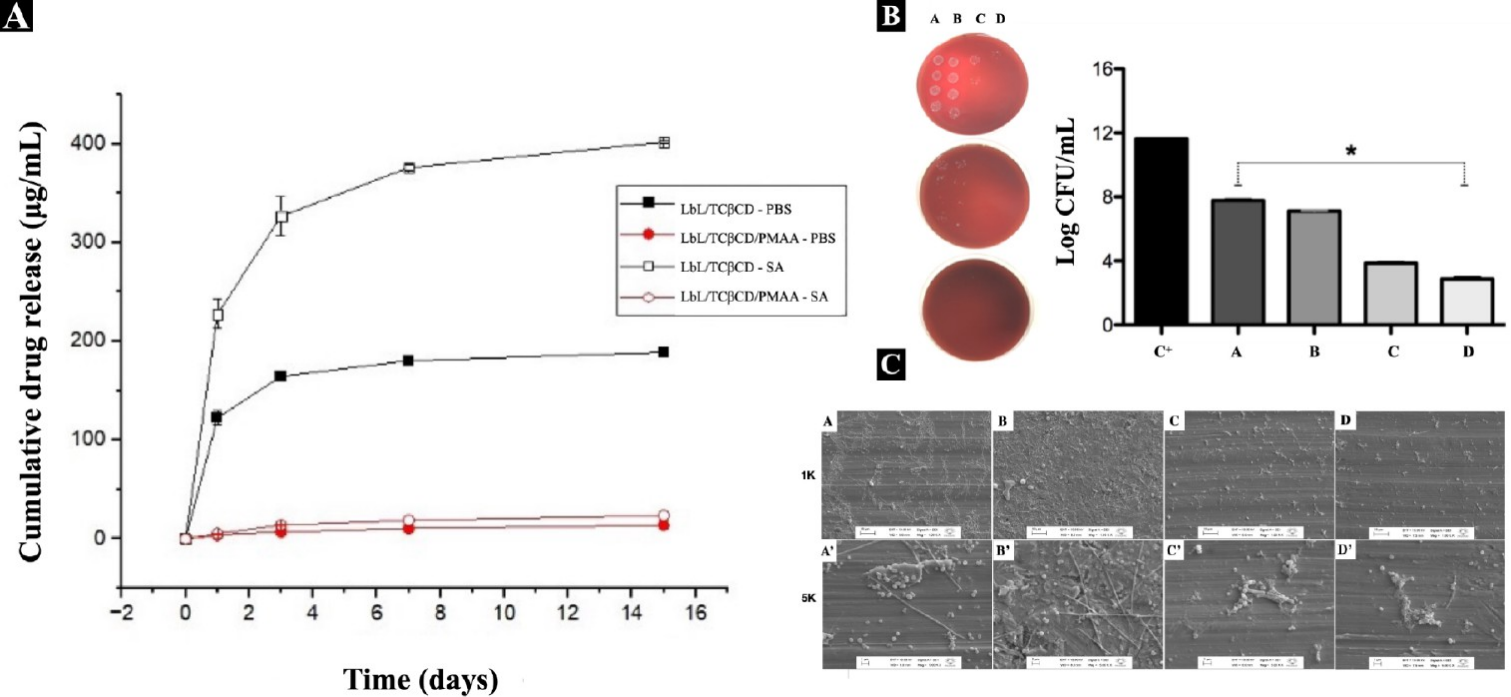
(A) Cumulative release profiles of LbL/TCβCD and LbL/TCβCD/PMAA-based films in PBS (pH 7.4) and acetate buffer (pH 4.5) at 37 °C. (B) Quantitative analysis of bacterial adhesion expressed as log CFU/mL, showing microbial growth from the saliva of subjects diagnosed with peri-implantitis on uncoated Ti, LbL/PMAA, LbL/TCβCD, and LbL/TCβCD/PMAA-coated Ti discs (C^+^: positive control; A: Ti; B: Ti/LbL/PMAA; C: Ti/LbL/TCβCD; D: Ti/LbL/TCβCD/PMAA). Statistical analysis was performed using one-way analysis of variance (ANOVA) followed by Tukey’s multiple comparisons post hoc test in GraphPad Prism version 10.10c (GraphPad Software, CA, USA). Data are presented as mean ± standard deviation (SD). Statistically significant differences are indicated as * *p* < 0.05 vs Ti control. (C) Representative images of microbial culture growth from peri-implantitis-derived saliva on control and experimental samples (A–A′: Ti; B–B′: LbL/PMAA; C–C′: LbL/TCβCD; D–D′: LbL/TCβCD/PMAA).

### 3.3 *In Vitro* Biological Approaches

#### 3.3.1 PMAA-Based Film Preserves the Antibacterial Activity of LbL/TCβCD

Building on our previous study, which confirmed the antibacterial activity of TC after complexation with βCD against *Staphylococcus aureus*—a common bacterial species associated with biofilms on medical devices and healthcare-associated infections^43,44^ —we investigated the broad-spectrum efficacy of TCβCD against a polymicrobial community from saliva of patients diagnosed with peri-implantitis. The antibacterial activity of the LbL/TCβCD/PMAA coating on Ti discs was evaluated using a standard colony-forming unit (CFU) analysis. After 3 days of incubation, the CFU count revealed a strong antimicrobial effect of the LbL/TCβCD coating, with nearly a 4-log reduction in bacterial growth compared to Ti discs (Figure 4B). Notably, the LbL/TCβCD/PMAA-based film exhibited an even stronger antimicrobial effect, suggesting that the superficial functional groups on the PMAA-based film effectively disturbed bacterial attachment across different classes of bacteria.

Qualitative analyses, including microbial community growth images, supported the CFU results, showing significantly reduced biofilm formation on LbL/TCβCD- and LbL/TCβCD/PMAA-coated surfaces compared to the Ti control group (Figure 4C). SEM observations of microbial community morphology revealed adherence of both spherical and bacilli structures regardless of the material surface. These findings corroborate previous findings, indicating that surface modifications can effectively reduce microbial adhesion and biofilm formation, which are critical factors in managing peri-implant infections.^41,42^

In fact, the antimicrobial activity of the drug delivery system is maintained through the synergistic effect of TC complexed with βCD (TCβCD) and the pH-sensitive PMAA-based film. The TCβCD complex exhibits broad-spectrum antibacterial activity, which has been previously demonstrated against *Staphylococcus aureus* (a common pathogen in medical device biofilms and peri-implant infections).^40^ In our study, the LbL/TCβCD coating significantly reduced bacterial colonization from polymicrobial saliva communities, achieving nearly a 4-log reduction in CFU counts compared to uncoated Ti discs (Figure 4B). The incorporation of the PMAA-based film further enhanced this effect, as the superficial functional groups of PMAA disrupted bacterial adhesion, thereby reducing biofilm formation. These properties collectively prevent recolonization of the surface, maintaining long-term antimicrobial activity and supporting the utility of this coating for managing peri-implant infections.

In this context, the review by Tiwari et al.^45^ emphasizes that antibiotic-releasing coatings, particularly those containing gentamicin, represent an effective strategy against bacterial adhesion and biofilm formation on implant surfaces. Such coatings provide localized delivery of antibiotics directly at the implant–tissue interface, reducing systemic toxicity and ensuring high local concentrations of the antimicrobial agent.^45^ However, despite their proven short-term efficacy, gentamicin-based systems are often associated with limitations, such as burst release, rapid depletion of the drug reservoir, and a consequent loss of long-term antimicrobial protection. Moreover, the uncontrolled release of antibiotics can contribute to the development of bacterial resistance, a critical concern in the context of chronic peri-implant infections.

#### 3.3.2 PMAA-Based Film Biocompatibility

According to the International Organization for Standardization (ISO) 10993, biocompatibility testing is a mandatory step in medical device development and regulatory approval, ensuring material safety and compatibility with biological systems. The interaction between cells and materials is influenced by material chemistry and topography, which affects cell adhesion and proliferation. Given that cell behavior can change rapidly, biocompatibility assessments are conducted over days to weeks. In this study, we evaluated the biological safety of the LbL/TCβCD/PMAA-based film coating on Ti discs using both epithelial and fibroblast cells cultured in monolayers and within 3D collagen matrices. Confocal microscopy images showed that the PMAA-based film did not adversely affect the proliferation and spreading of metabolically active cells. HaCaT (Figure 5A–D) and HGF (Figure 5E–H) cells demonstrated intact membrane integrity and comparable growth on the PMAA-coated surfaces compared to Ti control. The PMAA-based films exhibit a definitive pH-dependent swelling behavior that ensures controlled drug permeability. This characteristic allows the upper PMAA-based layer to effectively minimize local TC concentrations at the cell–material interface, thereby optimizing drug delivery and therapeutic outcomes.

**Figure 5:**
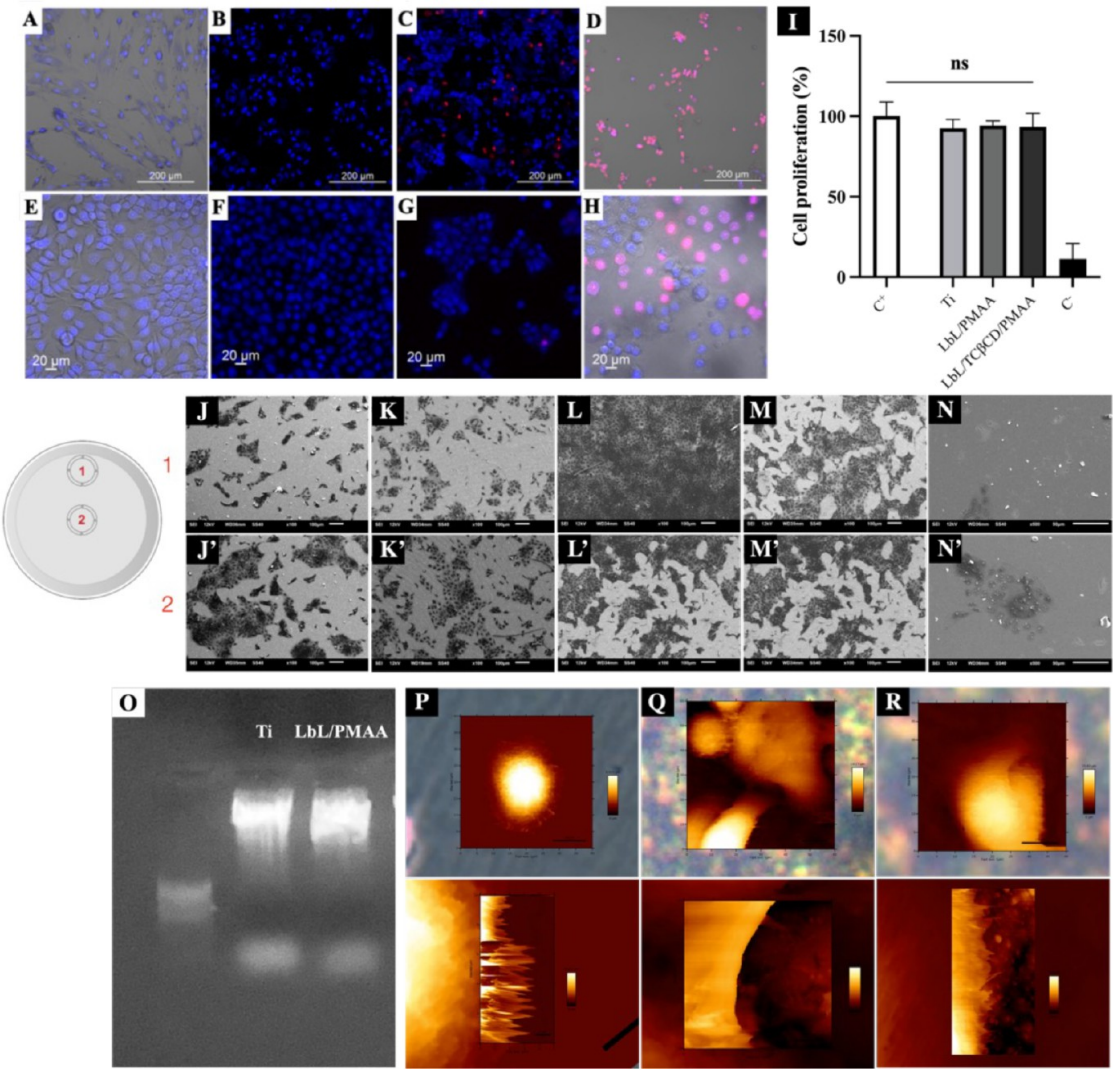
Evaluation of HaCaT (A–D) and HGF (E–H) cell behavior after 24 h of culture on different substrates. Live/dead staining images show viable cells in blue and dead cells in red on (A,E) the plate bottom, (B,F) Ti disc, (C,G) Ti disc coated with LbL/TCβCD/PMAA-based film, and (D,H) the negative control. (I) Effect of the LbL/TCβCD/PMAA-based film-coated Ti discs on HaCaT cell proliferation after 24 h of incubation (C^+^: positive control; C^–^: negative control). Statistical analysis was performed using one-way analysis of variance (ANOVA) followed by Tukey’s post hoc test. Data are presented as mean ± standard deviation (SD, *n* = 6), and differences were considered statistically significant at *p* < 0.05. Representative images of HaCaT cell morphology and spreading after 24 h on (J–J′) the plate bottom, (K–K′) Ti disc, (L–L′) Ti/LbL/PMAA, (M–M′) Ti/LbL/TCβCD/PMAA, and (N–N′) the negative control. (O) Agarose gel electrophoresis showing the absence of DNA fragmentation in the positive control group, indicating that the PMAA-based film coating did not compromise HaCaT DNA integrity. Images are representative of two independent experiments. AFM height images (scan size: 20 × 5 μm^2^) of living HaCaT cells cultured on (P) the plate bottom, (Q) the Ti disc, and (R) the Ti/LbL/TCβCD/PMAA-coated disc reveal cell morphology and surface interactions.

Our previous research^13,15^ confirmed the biocompatibility of the LbL system through both quantitative and qualitative assessments using HaCaT and HGF cells. Notably, we also assessed the cytotoxicity of the TCβCD concentration range (50 to 1 mg/mL). Although this previous study^15^ showed live cells upon exposure to all tested TCβCD concentrations, the highest concentration (1 mg/mL) showed initial signs of cellular damage, such as loss of membrane integrity marked by red fluorescent dots. In this study, with the LbL system loaded with 2 mg/mL TCβCD, potential impacts on cell viability by direct contact were considered. However, our results showed that the PMAA-based film provided protective effects, with HaCaT cells maintaining comparable viability across all experimental and control samples up to 24 h (Figure 5A–I).

Further investigation into the potential of the PMAA-based film to affect genomic integrity involved assessing DNA degradation. After 24 h of exposure, intact and well-defined DNA bands (red arrows) were observed in both the positive control (HaCaT cells cultured directly on the plate) and the LbL/TCβCD/PMAA-coated Ti discs, indicating that the film and its components did not adversely affect genomic DNA (Figure 5O). Multiple DNA bands would indicate degradation, which was not observed. As a limitation of this method, the absence of a fully validated negative control slightly constrains the confirmation of assay sensitivity; however, the consistent observation of intact DNA across all samples supports the conclusion that the coating does not induce detectable genotoxic effects

Considering that tissue surrounding implant abutment surfaces should form a biological seal to protect the implant from mechanical forces and microorganisms, we also evaluated the material’s ability to stimulate epithelial cell extension in real time (Figure 5P–R). AFM provided high-resolution topographical images under physiological conditions without extensive sample preparation. AFM images revealed differences in filopodial projections from epithelial cells on the positive control (bottom of the polystyrene plate), Ti (commercial implant surface), and LbL/PMAA-coated Ti discs. Filopodial projections at the cell edges indicate the affinity between cell molecules and the surface, suggesting potential cell adhesion. Additionally, SEM demonstrated a preference for epithelial cells to adhere to the coated surfaces compared to commercial Ti, with cells extensively covering areas near the edges of the LbL/PMAA-coated Ti discs (Figure 5J–N). These observations support the idea that surface modifications can significantly influence cellular behavior, aligning with literature on the role of functionalized surfaces in promoting favorable cell-surface interactions.^12,46^ The enhanced cellular response observed on LbL/PMAA-coated surfaces highlights their potential applications in implant design, where improved biocompatibility and tissue integration are critical for long-term success.

The biocompatibility of implant materials is critical for their successful integration into biological systems, particularly in applications such as dental implants.^47^ To further evaluate the safety of the PMAA-based film, we examined its effect on fibroblast-mediated collagen matrix contraction in an *in vitro* model over an extended period of 6 days. Our results showed that the PMAA-based film did not adversely affect mitochondrial activity at 1, 3, and 6 days of incubation, with no significant reduction compared to the live control (Figure 6A). In contrast, the death control exhibited a substantial decrease in cell metabolism, with more than a 60% reduction compared to the live control. Additionally, the PMAA-based film maintained the fibroblast’s ability to contract collagen gels throughout the 6-day incubation period, regardless of TCβCD incorporation (Figure 6B). These findings suggest that the PMAA-based film supports fibroblast activity and collagen matrix contraction, which are critical for effective wound healing and tissue integration.

**Figure 6:**
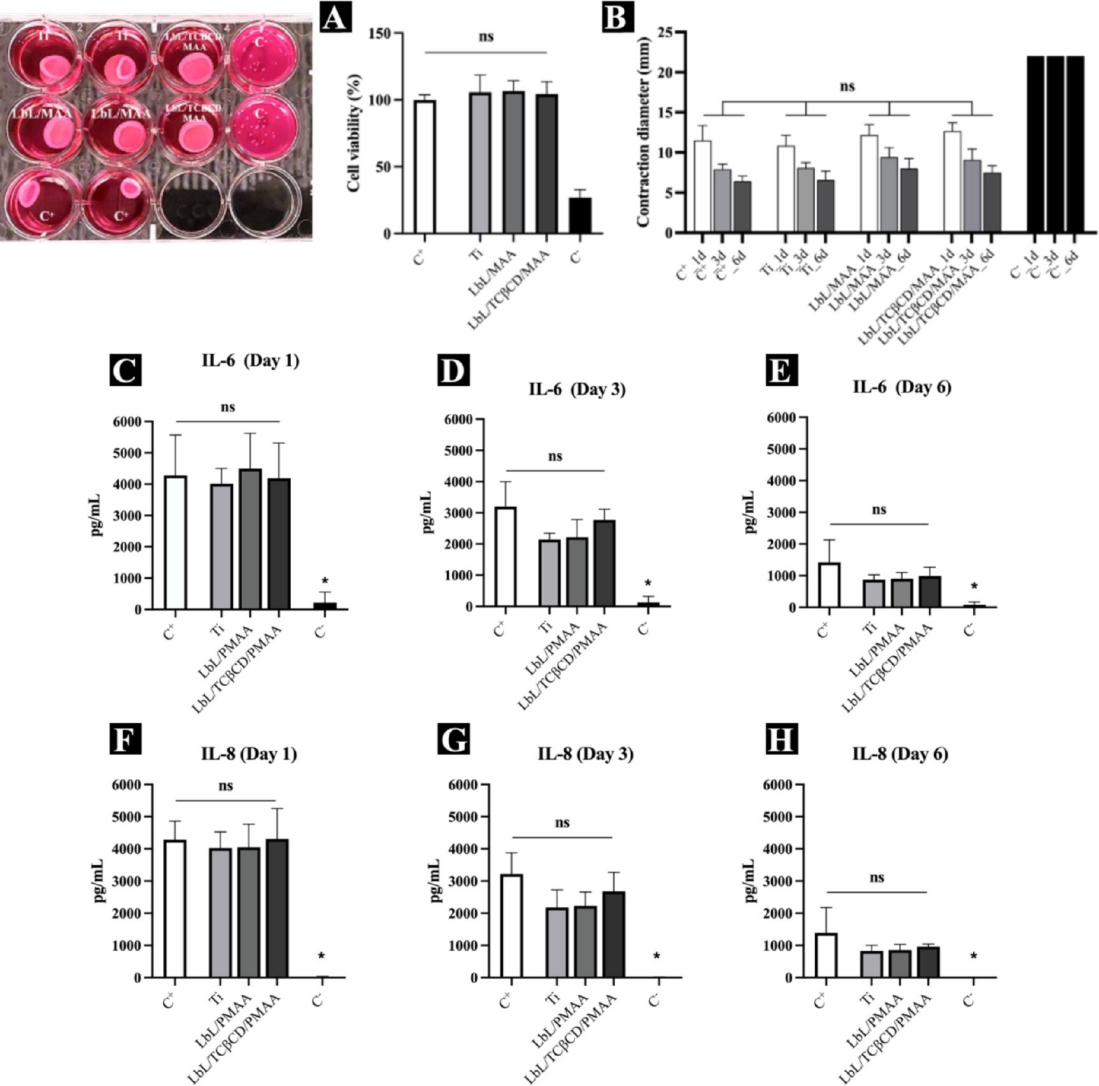
(A) Effect of LbL/TCβCD/PMAA-based film-coated Ti discs on the viability of HGFs within a collagen matrix after 1, 3, and 6 days of incubation, as assessed by the MTT assay (C^+^: positive control; C^–^: negative control). Statistical analysis was performed using one-way analysis of variance (ANOVA) followed by Dunnett’s multiple comparison post hoc test. Data are presented as mean ± standard deviation (SD, *n* = 6), with *p* < 0.05 considered statistically significant. (B) The effect of LbL/TCβCD/PMAA-coated Ti discs on collagen matrix contraction was quantitatively evaluated by measuring the contraction diameter and statistically analyzed using one-way ANOVA followed by Sidak’s multiple comparisons post hoc test. Data are expressed as mean ± SD (*n* = 6), and *p* < 0.05 was considered statistically significant. (C–E) Quantification of pro-inflammatory cytokine IL-6 expression after 1, 3, and 6 days of culture on the LbL/TCβCD/PMAA-coated Ti discs, assessed by one-way ANOVA with Dunnett’s multiple comparison post hoc test. (F–H) Quantification of IL-8 secretion under the same conditions. Data represent mean ± SD (*n* = 6), and statistically significant differences are denoted by *p* < 0.05.

The implantation of a biomaterial into the body triggers a natural inflammatory response, crucial for tissue healing. This localized inflammation facilitates leukocyte migration and the expression of inflammatory cytokines that mediate the pro-inflammatory response, followed by a resolution phase for effective healing.^48^ Dysregulation of this process can lead to uncontrolled inflammation, characterized by elevated levels of pro-inflammatory cytokines.^49,50^ To assess the impact of the PMAA-based film on the inflammatory response, we quantified pro-inflammatory cytokines released by HGF within a collagen matrix, comparing results with controls: cells cultured on the bottom of the plate and commercial Ti discs. Flow cytometry analysis revealed that HGF cells produced IL-6 and IL-8 at 1, 3, and 6 days (Figure 6C–H). No significant differences in cytokine levels were observed between the experimental and control groups, indicating that the PMAA-based film does not adversely affect the inflammatory response. Notably, statistical differences were found between the experimental groups and the negative control, showing that surface modifications did not increase secretion of inflammatory cytokines compared to the controls. These results underscore the potential of PMAA-based films to enhance cell adhesion while maintaining an appropriate inflammatory response, which is essential for successful tissue regeneration around implants. By modulating inflammation, PMAA-based films could contribute to the development of implants that minimize adverse reactions and promote effective healing.^41,42^

This 3D model was intentionally chosen because it more closely mimics the complex architecture and cell–matrix interactions of soft tissue, allowing for a more realistic evaluation of material biocompatibility and inflammatory response. Moreover, the selection of IL-6 and IL-8 was based on their well-established roles as early markers of inflammation and tissue response to biomaterials. IL-6 acts as a pleiotropic cytokine involved in both acute-phase inflammation and tissue regeneration,^51,52^ while IL-8 is a key chemokine responsible for neutrophil recruitment and the modulation of fibroblast activity at implant–tissue interfaces.^53–55^ Although a cytokine array was employed for initial screening, IL-6 and IL-8 were selected for quantitative evaluation due to their relevance in assessing the inflammatory behavior of fibroblasts in contact with titanium-based biomaterials. Other cytokines included in the array did not show significant modulation under the experimental conditions and therefore were not further analyzed in detail.

### 3.4 PMAA-Based Film Biocompatibility in Animal Model

All histological sections revealed similar connective tissue responses around subcutaneously implanted discs, regardless of whether they were coated with LbL/PMAA or LbL/TCβCD/PMAA. At day 2, we observed a delicate network of collagen fibers and an abundance of fibroblasts (Figure 7A–C). In addition, the presence of inflammatory cells and a dense network of blood vessels was noted (Figure 7A′–C′), indicative of an early inflammatory response conducive to tissue healing.^56,57^ The high density of blood vessels suggests a robust angiogenic response, which is critical for supplying nutrients and oxygen during the healing process.^58^ By day 7, there was an increase in collagen fiber density (Figure 7A′–C′) and a decrease in inflammatory cell presence, indicating a transition from the inflammatory phase to the proliferative and remodeling phase in the healing process.^50^ This observation is consistent with established theories that the initial inflammatory response is essential for tissue regeneration, while subsequent remodeling is crucial for long-term implant integration.^59^ The similarities in connective tissue characteristics across different coatings suggest that the PMAA-based films do not adversely affect the healing process. This is crucial for their potential clinical application. These findings indicate that the presence of LbL coatings supports an appropriate inflammatory environment conducive to tissue regeneration, aligning with studies that show how surface modifications can enhance biocompatibility and tissue integration.^12,46^ The observed patterns of connective tissue development also support literature emphasizing the importance of controlled inflammation for successful implant material integration.^58^

**Figure 7:**
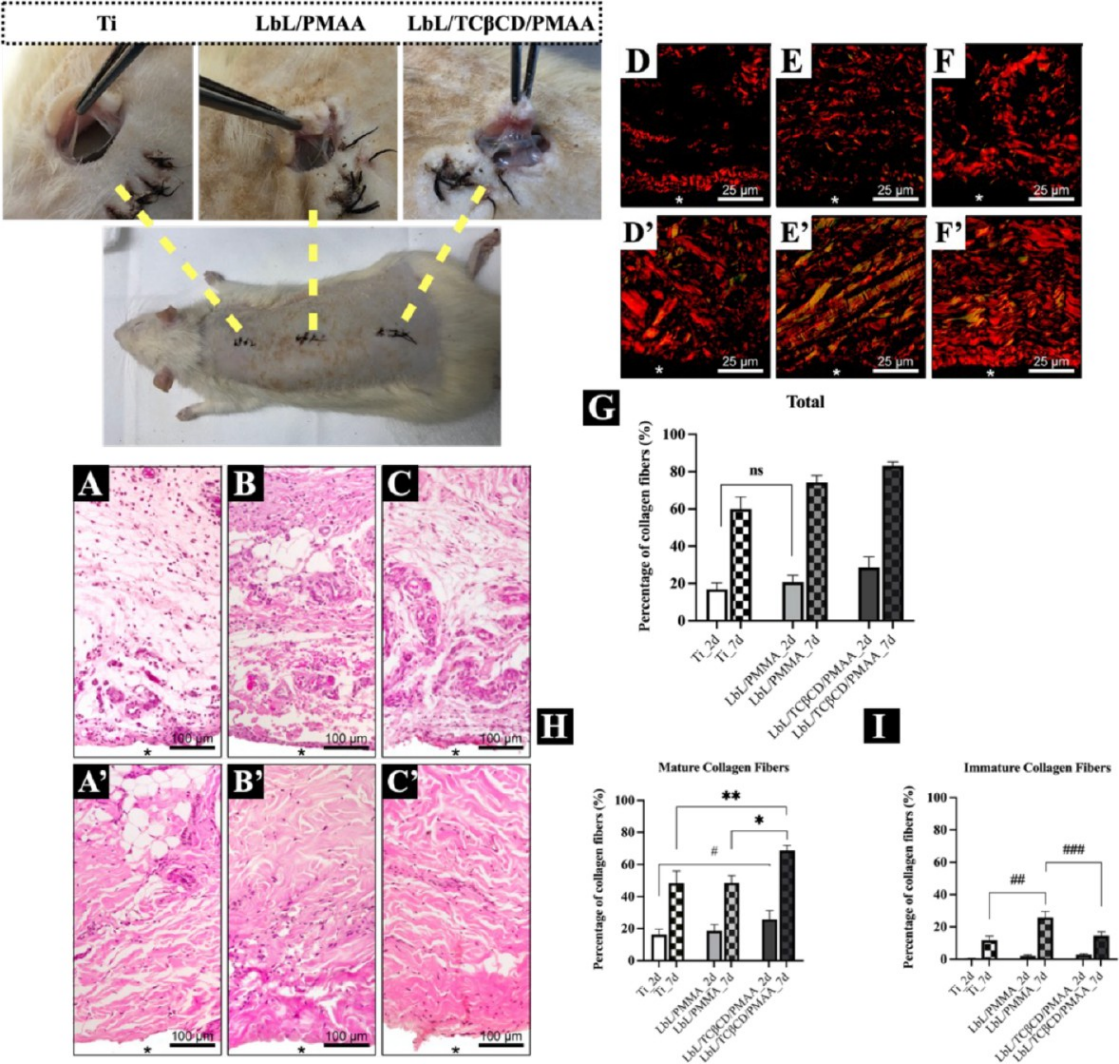
Histological analysis of connective tissue organization surrounding Ti, LbL/PMAA-, and LbL/TCβCD/PMAA-based film-coated Ti discs. Representative hematoxylin and eosin (H&E)-stained sections showing cellularity and structural organization around (A) Ti, (B) LbL/PMAA, and (C) LbL/TCβCD/PMAA coatings at (A–C) 2 days and (A′–C′) 7 days postoperatively. Asterisks indicate the space previously occupied by Ti discs. Magnification: 200×; scale bars: 100 μm. Picrosirius red-stained sections illustrating collagen fiber distribution and maturation around (D) Ti, (E) LbL/PMAA, and (F) LbL/TCβCD/PMAA coatings at (D–F) 2 days and (D′–F′) 7 days postoperatively. Quantitative assessment of total (G), mature (H), and immature (I) collagen fiber content was performed using one-way analysis of variance (ANOVA) followed by Šídák’s multiple comparison post hoc test. Data are expressed as mean ± standard deviation (SD), and statistically significant differences are indicated as *, **, #, ##, ### (*p* < 0.05).

Since the fine-tuning of collagen fiber assembly in the connective tissue also appears as a strategy to enhance soft tissue integration, the maturation level, organization, and amount of collagen fibers in tissues surrounding Ti, LbL/PMAA-based film, and LbL/TCβCD/PMAA-based film surfaces were investigated by picrosirius red under polarized light microscopy (Figure 7D–F′). After the first 48 h, tissue surrounding LbL/PMAA discs demonstrated a slight difference in the total amount of collagen fibers compared with the Ti control (Figure 7D–F). However, this little difference was suppressed after 7 days, with both qualitative and quantitative assessments revealing a clear increase in the amount of collagen fibers in the tissue surrounding LbL/PMAA and LbL/TCβCD/PMAA discs (Figure 7D–I). Conveniently, LbL/TCβCD/PMAA discs affected the total amount of collagen fibers independently of the time point evaluated. The birefringence pattern of collagen fibers using the aforementioned technique is also applied to distinguish immature (green-yellow) and mature (orange-red) collagen fibers. Interestingly, LbL/TCβCD/PMAA-based film surfaces presented significantly higher amounts of mature collagen fibers, while the Ti controls presented a higher amount of immature collagen fibers after 7 days (Figure 7I′). Correlating these data with the total amounts of collagen fibers observed in these two groups, it seems reasonable to conclude that there was acceleration of the maturation of collagen fibers in contact with the LbL/TCβCD/PMAA-based film. Although *in vitro* investigations have reported an apparent effect of TC on osteoclastogenesis,^60^ the actual role of this drug in bone remodeling is largely unknown. With regard to the mechanism behind the action of TC on the fibrillogenesis and maturation of collagen, whether directly or indirectly, it still needs to be unraveled.

It is important to highlight that these analyses represent a preliminary *in vivo* investigation conducted in a subcutaneous animal model to evaluate early connective tissue responses to LbL-based coatings. Histological sections showed similar inflammatory patterns across groups, characterized by a transient inflammatory phase followed by tissue remodeling, confirming that the PMAA-based films did not impair the healing process. These findings indicate that the coating may contribute to a more structured and resilient soft tissue interface, which is crucial for maintaining peri-implant stability. The increase in mature collagen fibers resembles the biological outcomes seen in connective tissue grafting procedures, where greater tissue thickness enhances protection against inflammatory processes and promotes long-term implant success. While these observations should be interpreted with caution given the preliminary animal model, they support the hypothesis that LbL/TCβCD/PMAA films may create a biological environment favorable to fibroblast activity and collagen maturation. This aligns with reports demonstrating that controlled inflammation, angiogenesis, and extracellular matrix organization are critical for improving soft tissue sealing and implant integration.^47,49,50^

From a clinical perspective, the increased amount of collagen fibers observed seems favorable to the process of connective tissue grafting, where the primary goal is to gain tissue thickness rather than keratinization.^61^ Just as in these grafts, where the focus is on achieving a thicker, more fibrous tissue to enhance implant protection, the presence of more collagen fibers would increase the resistance to inflammatory processes. While we cannot conclusively state that these fibers are directly inserted into the film based on this analysis, this does not diminish the significance of the result. The increased quantity of mature fibers contributes to greater tissue strength and resilience, which parallels the clinical objective of connective tissue grafts to fortify and thicken the tissue, thereby offering enhanced protection and resistance around the implant site.

Although no direct evidence has yet confirmed the impact of increased collagen fibers on disease prevention, the connective tissue graft is widely used in dental reconstructive surgery to convert thin to thick soft tissue. This implies a higher density of fibroblasts and, consequently, more collagen fibers, providing greater tissue resistance to inflammatory processes. The findings by Górski et al.^61^ support this assumption, as their randomized, split-mouth, double-masked, controlled clinical trial over 12 months demonstrated an overall increase in the number of collagen fibers in tissues treated with hyaluronic acid in addition to subepithelial connective tissue graft, compared with connective tissue graft alone, for the treatment of multiple gingival recession areas compared to normal mucosal connective tissue.^61^ This was expected due to the regenerative processes following surgical intervention, which led to scar tissue formation. Moreover, they observed a trend of increased collagen density in the study group compared with the control group. Therefore, the observed increase in collagen fibers, both in quantity and orientation, is a favorable outcome, reflecting a scenario similar to clinical regeneration procedures where tissue thickness enhances implant protection and resilience against inflammation. This emphasizes the importance of connective tissue responses in promoting healing and stability around implants, even though further research is needed to clarify the integration of the coating films in this process.

It is essential to consider the potential influence of the injury environment on the local functions of neutrophils and macrophages. Macrophages can be classified into two functionally distinct subtypes: (1) classically activated M1 macrophages, induced by cytokines such as IFN-γ, TNF-α, IL-1β, and IL-6, which exhibit pro-inflammatory, antibacterial, and antiviral activities, and (2) alternatively activated M2 macrophages, stimulated by IL-4 and IL-13, which possess anti-inflammatory and tissue repair/regeneration functions and express high levels of IL-10.^55,62–64^ To further investigate the inflammatory response surrounding the subcutaneously implanted discs, immunohistochemistry was performed to evaluate the protein levels of specific resident cells, including polymorphonuclear neutrophils (Figure 8A–F), M1 macrophages marked by iNOS expression (Figure 8G–L), and M2 macrophages marked by CD206 expression (Figure 8M–R). On day 2, a notable increase in the inflammatory response was observed, characterized by elevated neutrophil (CD45) expression across all groups compared to the levels seen at day 7 (Figure 8S). Conversely, the protein levels of monocytes (Figure 8T) were higher on day 7 than on day 2 in all groups (Figure 8T). Interestingly, the number of monocytes in the LbL/TCβCD/PMAA group was significantly lower than in the Ti group at day 7. The expression of M1 macrophages decreased from day 2 to day 7 across all groups, with a trend indicating a more pronounced reduction in the LbL/TCβCD/PMAA group compared with the other groups (Figure 8U). Importantly, the levels of M2 macrophages, which serve as anti-inflammatory markers, were significantly higher in the LbL/TCβCD/PMAA group than in all other groups on day 7, highlighting the anti-inflammatory potential of this treatment (Figure 8V).

**Figure 8:**
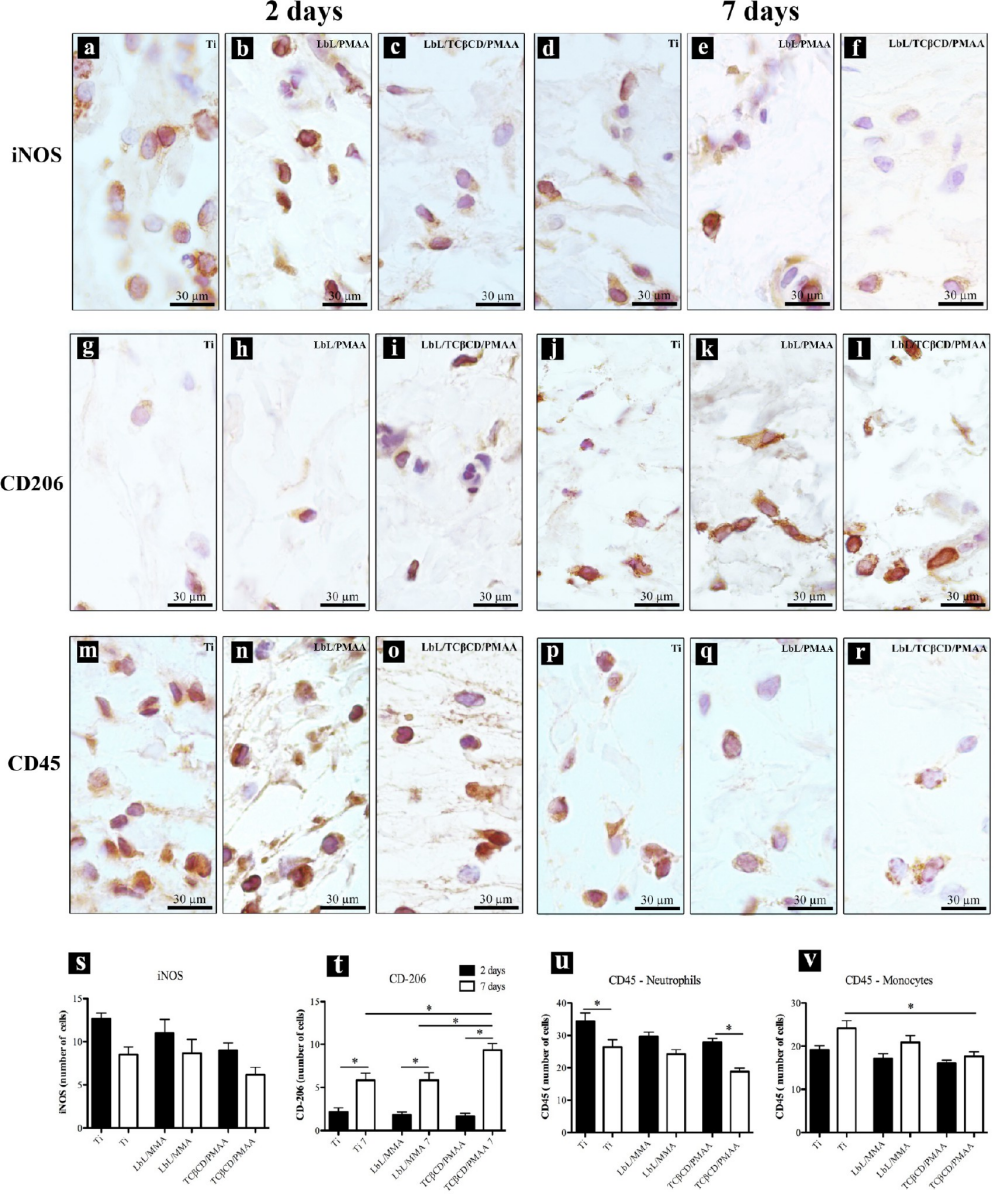
Immunolabeling analysis of inflammatory and macrophage cell populations within the connective tissue surrounding Ti, LbL/PMAA-, and LbL/TCβCD/PMAA-coated Ti discs. (a–d) Quantitative analysis of CD45^+^ polymorphonuclear leukocytes (a), CD45^+^ mononuclear leukocytes (b), M1 macrophages (iNOS^+^) (c), and M2 macrophages (CD206^+^) (d) in the different experimental groups. Statistical comparisons were performed using one-way analysis of variance (ANOVA) followed by Tukey’s multiple comparison post hoc test (GraphPad Prism version 10.10c, GraphPad Software, CA, USA). Data are presented as mean ± standard deviation (SD). Representative photomicrographs showing CD45^+^ polymorphonuclear and mononuclear leukocytes in the connective tissue for (e,h) Ti, (f,i) LbL/PMAA, and (g,j) LbL/TCβCD/PMAA groups at 2 (e–g) and 7 (h–j) days postoperatively. Immunolabeling of M1 (k–p) and M2 (q–v) macrophages in the connective tissue surrounding (k,n,q,t) Ti, (l,o,r,u) LbL/PMAA, and (m,p,s,v) LbL/TCβCD/PMAA at 2 (k–m,q–s) and 7 (n–p,t–v) days postoperatively. Magnification: 2000×; scale bars: 30 μm.

The observed increase in mature collagen and M2 macrophages in the LbL/TCβCD/PMAA group is likely an indirect consequence of a more regulated inflammatory environment, driven by the antimicrobial action of tetracycline rather than a direct effect of the drug on collagen fibrillogenesis. The enhanced collagen maturation observed in the presence of the LbL/TCβCD/PMAA coating thus appears to result from improved infection control and a more favorable microenvironment for fibroblast activity rather than direct biochemical stimulation of extracellular matrix synthesis. This interpretation aligns with previous evidence showing that antibiotic-releasing coatings modulate tissue responses primarily through infection control, indirectly promoting tissue organization and repair.^15^

Notably, lesions from human periodontitis and peri-implantitis predominantly display the M1 macrophage subset, accompanied by increased expression of pro-inflammatory mediators such as TNF-α, IL-1β, IL-6, and MMP-9.^64–67^ Additionally, recent studies on peri-implantitis lesion samples have identified a significant rise in M1 macrophages.^66,67^ These macrophages play a key role in driving the pro-inflammatory response triggered by bacterial presence, while M2 macrophages are involved in resolving inflammation and promoting tissue repair.^66,67^ These findings underscore the significance of our data, particularly the observed increase in M2 macrophage levels. This shift may play a crucial role in mitigating inflammation during peri-implantitis progression, potentially slowing the advancement of the lesion and promoting better tissue outcomes when the LbL/TCβCD/PMAA coating is used. Finally, it is important to note that macrophage polarization in inflammatory lesions may not only account for the contrasting immunoregulatory properties of M1 and M2 but also for their differential potential as osteoclast precursors.^62^ Specifically, the M1 subset has a greater osteoclastogenic differentiation capacity compared to M2 cells.^68^ Temporal analyses of inflammation progression to the healing of periodontal and peri-implant lesions have shown a shift from M1 macrophage activation (CD80 and TNF-α expression) to the M2 phenotype (CD206 expression), which correlates with alveolar bone loss.^66,67,69^

Although the subcutaneous implantation model does not replicate the infection dynamics of peri-implantitis, it provides critical insight into the biocompatibility and local tissue response to the coating. The antibacterial performance of the LbL/TCβCD/PMAA system was rigorously assessed *in vitro* using a polymicrobial biofilm derived from saliva of peri-implantitis patients, representing a clinically relevant infection model. The significant reduction in bacterial adhesion observed even after 48 h supports the coating’s antibacterial potential. Future work by our group is expanding this evaluation to an *in vivo* infection model to confirm the system’s efficacy in a biofilm-driven environment

## 4 Conclusion

In summary, the PMAA-based film on our drug delivery system emerges as a promising strategy to enhance current clinical outcomes, serving as an effective adjuvant in the management of peri-implantitis. The stimuli-responsive film stands out as a highly innovative triple-function coating:

Showed a noncytotoxic effect over the study period in both monolayer cultures and collagen matrix environments.
Demonstrated the ability to manage drug release even under neutral conditions. Responding to environmental changes, particularly acidic pH in inflamed areas, ensures precise and timely delivery of antimicrobial agents.
Not only maintained the antimicrobial activity of the drug delivery system but also improved it by sustaining the strong antibacterial properties of the associated drug delivery system, showing its potential to prevent the recolonization of the surface by microorganisms.
Promoted a favorable cell behavior at the soft tissue-abutment interface, supporting biological sealing and reducing the risk of infection.

Therefore, these features make the stimuli-responsive film, especially when combined with the LbL system, an advanced and tailored coating option for abutment surfaces. This targeted strategy could significantly improve the management of infectious-inflammatory conditions around dental implants, ultimately enhancing the durability and success of implant-supported treatments.

## Supplementary Material



## Notes

Analytical data confirming the successful synthesis and reproducibility of anionic β-cyclodextrin (βCD), the tetracycline−βCD complex (TCβCD), and poly­(methacrylic acid) (PMAA) (PDF)

## Abbreviations

PMAA: Poly­(methacrylic acid)
TC: Tetracycline
TCβCD: Tetracycline−β-cyclodextrin complex
βCD: Beta-cyclodextrin
LbL: Layer-by-layer assembly
Ti: Titanium disc
PBS: Phosphate-buffered saline
SA: Sodium acetate buffer
ζ Potential: Zeta (ζ) potential measurements
HaCaT: Immortalized human keratinocytes
HGF: Human gingival fibroblasts
IL: Interleukin
IL-6: Interleukin 6
IL-8: Interleukin 8
IL-1β: Interleukin 1β
IL-10: Interleukin 10
IL-12: Interleukin 12
TNF-α: Tumor necrosis factor-alpha
AFM: Atomic force microscopy
SEM: Scanning electron microscopy
C^+^: Positive control
C^–^: Negative control
ISO: International Organization for Standardization
XPS: X-ray photoelectron spectroscopy
PLL: Poly l-lysine
PEI: Polyethylenimine
ECH: Epichlorohydrin
CAA: Chloroacetic acid
TEMED: 1,2-Bis­(dimethylamino)­ethane
MAA: Methacrylic acid
AA: Acrylic acid
Allyl: Allylamine
FTIR: Fourier transform infrared spectroscopy
ATR: Attenuated total reflectance
CFU: Colony-forming unit
ISO: International Organization for Standardization
IFN-γ: Interferon-gamma
