# Supplementary material for: Targeted Infection Control and Tissue Integration via pH-Sensitive Smart Coatings on Implant Surfaces
Source: ACS Appl Mater Interfaces. 2025 Dec 23;18(1):2584–603. doi: 10.1021/acsami.5c19344 (PMC12781112; doi:10.1021/acsami.5c19344)
Supplement: Supplementary file 1 [file am5c19344_si_001.pdf]

Supporting Information

Targeted Infection Control and Tissue Integration via pH-Sensitive Smart Coatings on Implant Surfaces

Marta Maria Alves Pereira[1,2], Rodolfo Debone Piazza[3], Paula Aboud Barbugli[1], Oya Tagit[4], Jeroen J.J.P. van den Beucken[5], Abhijna Das[4], Cleyton Alexandre Biffe[6], Valentim Adelino Ricardo Barão[7], Stéfany Barbosa Alves da Cruz, Edilson Ervolino[9], Leonardo Perez Faverani[10], Vinicius Franzao Ganzaroli[9], Daniela Leal Zandim-Barcelos[11], Magda Feres[12], Belén Retamal-Valdes[13], Denise Madalena Polimari Spolidorio[14], Ana Claudia Pavarina[1], Amanda Paino Santana[8], Beatriz Severino Verza[1], Rodrigo Fernando Costa Marques[3], Rafael Scaf de Molon[8], Erica Dorigatti de Avila[1,8,*]

[1]Department of Dental Materials and Prosthodontics, São Paulo State University (UNESP), School of Dentistry, Araraquara, São Paulo, 14801-903, Brazil

[2]Postgraduate Program of Dentistry, Federal University of Piauí, Teresina, Piauí, 64049-550, Brazil

[3]Department of Analytical Chemistry, Physical Chemistry and Inorganic, Institute of Chemistry, São Paulo State University (UNESP), Araraquara - SP, 14800-060, Brazil.

[4]Institute of Chemistry and Bioanalytics, School of Life Sciences. University of Applied Sciences and Arts Northwestern Switzerland. Muttenz, 4132, Switzerland

[5]Dentistry - Regenerative Biomaterials, Radboudumc, Philips Van Leydenlaan 25, Nijmegen, the Netherlands

[6]Brazilian Nanotechnology National Laboratory (LNNano), Brazilian Center for Research in Energy and Materials (CNPEM), Campinas, São Paulo, 13083-970, Brazil.

[7]Department of Prosthodontics and Periodontology, Universidade Estadual de Campinas (UNICAMP), Faculdade de Odontologia de Piracicaba (FOP), Piracicaba, São Paulo, 13414-903, Brazil

[8]Department of Diagnosis and Surgery, São Paulo State University (UNESP); School of Dentistry, Araçatuba, São Paulo, 16015-050, Brazil.

[9]Department of Basic Sciences, São Paulo State University (UNESP), School of Dentistry, Araçatuba, São Paulo, 16018-805, Brazil

[10]Department of Oral Diagnosis, Division of Oral and Maxillofacial Surgery, University of Campinas (UNICAMP), Piracicaba Dental School, Piracicaba, São Paulo, 13414-903, Brazil; Postgraduate

supervisor on OMFS at the São Paulo State University (UNESP); School of Dentistry, Araçatuba, São Paulo, 16015-050, Brazil.
[11]Department of Diagnostic and Surgery, São Paulo State University (UNESP), School of Dentistry, Araraquara, São Paulo, 14801-903, Brazil
[12]Department of Oral Medicine, Infection, and Immunity, Harvard School of Dental Medicine, Boston, Massachusetts, 02115, USA
[13]Department of Periodontology, RS Master Saude, Aruja, São Paulo, 07400445, Brazil
[14]Department of Physiology and Pathology, São Paulo State University (UNESP), School of Dentistry, Araraquara, São Paulo, 14801-903, Brazil

Corresponding author*
Dr. Erica Dorigatti de Avila
Department of Diagnosis and Surgery
São Paulo State University (UNESP), School of Dentistry
Rua José Bonifácio, 1193. Araçatuba, São Paulo, 16015-050, Brazil
Phone: +55 16 997994002
Email: erica.avila@unesp.br

Results & Discussion

Successful reproduction of anionic βCD, TCβCD and PMAA

We have successfully demonstrated that anionic βCD, a cyclic oligosaccharide with a hydrophobic inner cavity and hydrophilic hydroxyl groups on its outer surface, plays a crucial role in enhancing drug loading capacity and controlling drug release. To confirm the reproducibility of anionic βCD synthesis, we performed NMR analysis. Figure 1S-A shows the ^{1}H NMR spectra of anionic βCD recorded in deuterated solvent, compared to our previous results (Fig. 1B). Interpretation of the ^{1}H 1D spectrum indicates that each glucose ring has one chloroacetic acid moiety attached. This suggests that epichlorohydrin was non-reactive. The heteronuclear multiple bond correlation (HMBC) to ^{13}C at 177 ppm confirms the presence of chloroacetic acid, identified by the C–O chemical shift. These results demonstrate that the negative charge from the anionic molecules is incorporated into the hydrophilic group extending from the wider opening of the βCD. These findings are consistent with our previous study [16] that highlight the advantages of using cyclodextrins in drug delivery systems (Fig. 1S-B). For example, research has shown that cyclodextrin incorporation can significantly enhance the solubility and stability of poorly water-soluble drugs. In our study, the anionic modification of βCD improved interaction with drug molecules, leading to higher drug-loading efficiency and more controlled release profiles.

The incorporation of TC into anionic βCD was analyzed using FTIR spectroscopy, as shown in Figure 1S-C. The spectrum of anionic βCD reveals a band at 1730 cm^{-1}, corresponding to the carboxyl (C=O) modification of βCD. Key features also include the overtone bending of methylene (C-H) groups around 1465 cm^{-1} and the ether (C-O-C) stretches from the glucose units at 1015 cm^{-1}. For the TC sample, characteristic bands were observed, including the N-H bending of amide or amine groups in the range of 1613–1519 cm^{-1}. A band at 1229 cm^{-1} was attributed to the C-N bond stretching of the tertiary amine. Additionally, methylene and methyl group bending absorptions were detected at 1452 cm^{-1} and 1309 cm^{-1}, respectively, while out-of-plane bending of the aromatic C-H bond appeared at 860 cm^{-1} and 691 cm^{-1}. The spectra of both anionic β-CD and the TC/anionic β-CD complex were similar, with slight shifts due to the presence of TC bands at 1613, 1447, 1229, and 691 cm^{-1}. These findings strongly support the successful complexation of the drug with the polymer [16].

Finally, the successful synthesis and development of PMAA were also confirmed by FTIR spectroscopy.

As shown in Figure 1S-D, the bands between 3000 and 2850 cm⁻¹ correspond to C-H stretching of alkanes. The band at 1740 cm⁻¹ is characteristic of the carbonyl (C=O) group present in acrylic acid and methyl acrylic acid moieties. The band at 1224 cm⁻¹ indicates C-N stretching of the allyl amine, while the band at 1364 cm⁻¹ corresponds to C-H bending. The absence of bands between 1648 and 1638 cm⁻¹, which refer to the vinylic group (C=C) present in all monomeric structures, confirms the successful synthesis of the copolymer.

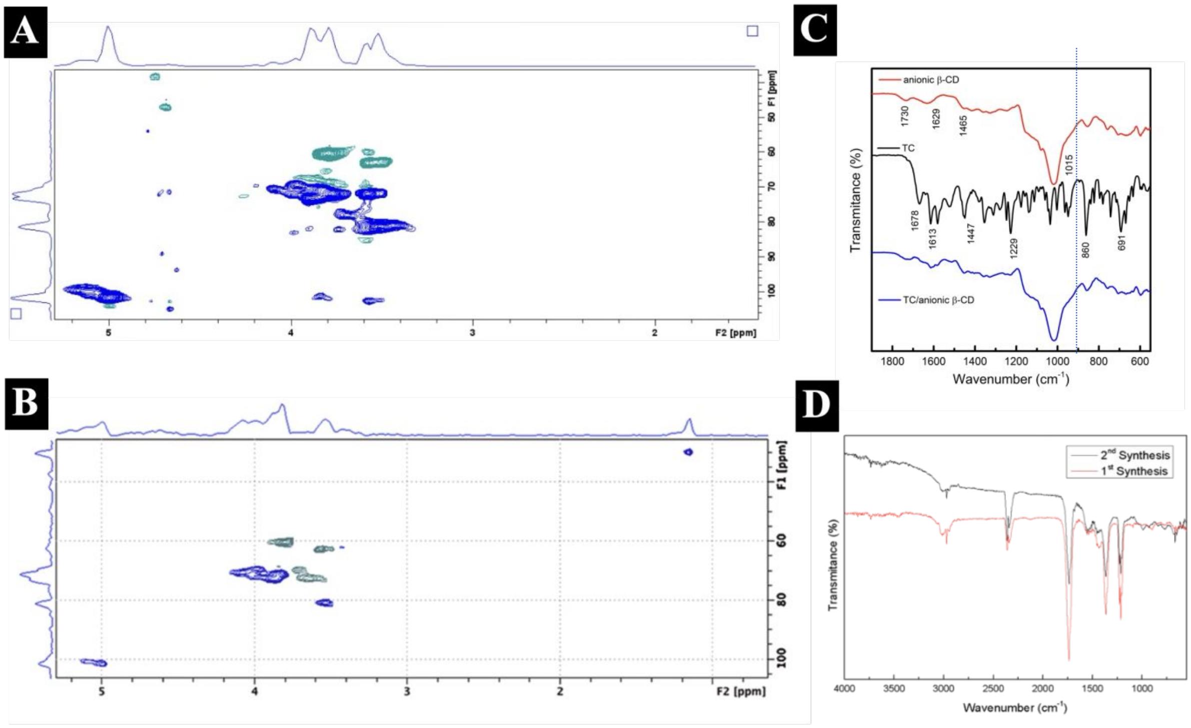

Figure S1. (A) 1H NMR spectra of βCD 1 mM, anionic βCD 1 mM in D_2O from the synthesis performed to study in question. (B) 1H NMR spectra of βCD 1 mM, anionic βCD 1 mM in D_2O described by Verza et al., 2021. (C) Diffraction peak data of TC, anionic βCD and TC/βCD crystal measured in parallel beam mode (parabolic mirror). Note the change in intensity for each material (D) FTIR spectra of two PMAA-based film synthesis, to confirm the reproducibility in the material production in different occasions. Figure B has been reprinted and adapted with permission from Elsevier (License Number: 5881521482532).
